# Enhanced SynPID by Tracking Varying Targets Applied to General Anaesthesia

**DOI:** 10.3390/bioengineering13101199

**Published:** 2026-10-15

**Authors:** Carlos E. Castañeda, Onofre Orozco-López, Alfredo Abad-Gurumeta, Agustín Rodríguez-Herrero

**Affiliations:** 1 Universidad de Guadalajara, Centro Universitario de los Lagos, Departamento de Ciencias Exactas y Tecnología, Av. Enrique Díaz de León No. 1144, Colonia Paseos de la Montaña, Lagos de Moreno 47460, Jalisco, Mexico; carlose.castanedah@academicos.udg.mx; 2 Department of Anesthesiology, Infanta Leonor Hospital, 28031 Madrid, Spain; alfredo.abad@salud.madrid.org; 3 Departamento de Telemática y Electrónica, Escuela Técnica Superior de Ingeniería y Sistemas de Telecomunicación, Universidad Politécnica de Madrid, 28031 Madrid, Spain; agustin.rodriguez@upm.es

**Keywords:** closed-loop anaesthesia, ESynPID, PID inversion, symmetric weighting, target tracking, propofol, remifentanil, rocuronium

## Abstract

Closed-loop anaesthesia systems use automated controllers to regulate drug delivery and improve anaesthetic management. Although PID-based strategies have shown promising performance, challenges related to patient variability, robustness, setpoint definition, and clinical implementation remain open. This work presents an enhanced synergistic PID strategy, denoted as ESynPID, for multivariable drug delivery in general anaesthesia. The proposed approach extends the previous SynPID formulation by incorporating a variable setpoint generation mechanism through a PID Inversor, a symmetric weighting strategy for the setpoint feedforward terms, and an updated symmetric control-to-range module. The virtual patient is represented through pharmacokinetic–pharmacodynamic models for propofol, remifentanil, and rocuronium, numerically propagated in a sampled-data framework. The resulting architecture combines tracking-oriented target generation, weighted errors PID control, range correction, open-loop/closed-loop phase switching, and a pump emulation block including safety, quantification, and zero-order hold stages. The proposed controller is evaluated in a cohort of virtual patients under surgical stimulation scenarios and compared with the previous SynPID approach. The results show that ESynPID modifies the perfusion profiles by anticipating drug delivery around stimulus changes and by producing more active tracking behaviour during the maintenance phase. This improves the regulation of the hypnotic monitor, while revealing a trade-off in the nociception response. Overall, the study shows that the proposed tracking and symmetry mechanisms increase the adaptability of the control law, although their effect depends on the monitored variable and on the interaction between the weighting strategy, the inversion mechanism, and the pharmacokinetic dynamics of the patient.

## 1. Introduction

A protocol in total intravenous anaesthesia is a set of drug infusions and boluses whose goal is to maintain a patient in an adequate anaesthetic state. If the clinician makes the decisions about modifying the protocol, it is referred to as an open-loop protocol (OLP). When decisions are made using algorithms, the protocol is referred to as a closed-loop protocol (CLP); these algorithms must be supervised by an anaesthetist. CLP protocols offer numerous benefits, such as reducing subjectivity in the anaesthetist’s decision-making regarding drug administration, minimising medication usage, reducing patient recovery time, and enabling a single anaesthetist to supervise multiple surgeries simultaneously from a control room [1].

During the twenty-first century, CLP systems based on PIDs (proportional, integral and derivative controllers), artificial intelligence and fuzzy logic are being developed, featuring feedback based on bispectral index (BIS), pain by nociception index (NOX) and/or relaxation on neuromuscular blockade (NMB) monitors. A PID is a controller that provides a control law governed by an error signal, derived from the deviation between the controlled variable and a reference signal or set point. The control law consists of proportional, integral and derivative actions applied to the error signal [1]. Recent research in closed-loop anaesthesia has increasingly focused on PID-based strategies for co-administering multiple intravenous drugs while tracking physiological variables such as hypnosis, analgesia, and neuromuscular blockade. Building on early event-based and ratio-adjustable PID formulations, more recent developments emphasise multivariable structures capable of handling drug interactions and varying clinical targets [2]. Castañeda et al. [2] developed SynPID, a personalised asymmetric MIMO-PID (SynPID) controller for the co-administration of propofol, remifentanil and rocuronium. Feedback was guided using monitors of hypnosis by BIS, NOX and NMB. Also, SynPID incorporates feedforward modulation, control to range and safety constraints. In virtual testing, this architecture improved disturbance rejection and maintained satisfactory anaesthesia through patient-specific automatic tuning.

These advances complement earlier contributions by Paolino et al. [3], who proposed an event-driven PID to reduce unnecessary actuator updates, and by Schiavo et al. [4], who experimentally demonstrated that tunable opioid–hypnotic ratios enhance clinical flexibility. Together, these studies show a clear trend toward enhanced multivariable PID formulations capable of adapting to drug synergies, patient-specific dynamics, and varying therapeutic targets. Such developments provide a strong foundation for the proposed Enhanced SynPID, which aims to further improve target tracking under dynamic anaesthetic conditions.

Kagami et al. [5] proposed a multiobjective optimisation framework using decentralised PID controllers for safe drug delivery during the hypnotic induction phase, balancing variables like pain perception and mean arterial pressure. However, the PID controllers showed performance limitations during subsequent surgical stages. Furthermore, the complexity of the multiobjective optimisation process and the challenges in conducting trade-off analyses in the obtained Pareto Front may hinder practical implementation in clinical settings. Padula et al. [6] utilised genetic algorithms and gain scheduling to optimise a PID controller for anaesthesia induction and maintenance. Although the approach improves induction time and disturbance rejection, it suffers from performance degradation if parameters are not dynamically adjusted between phases. Furthermore, managing BIS signal noise with a low-pass filter introduces control trade-offs, and the system still faces challenges regarding patient variability. Muresan et al. [7] introduced a fractional-order controller for depth of analgesia based on skin impedance and remifentanil administration, offering higher flexibility than integer-order alternatives. Despite its accuracy, the approach faces limitations regarding data generalisability and the clinical implementation challenges of complex fractional-order models. Additionally, robust tuning is still necessary to overcome individual patient variability.

Some works have explored the regulation of the opioid–hypnotic balance through an optimised PID control scheme for the co-administration of propofol and remifentanil. Emphasising the importance of maintaining a fixed infusion ratio that can be adjusted by the clinician, demonstrating that a well-tuned PID controller can effectively manage the synergistic effects of drugs on the bispectral index (BIS) and hemodynamic stability [8]. While other ones designed a fractional-order PID (FOPID) controller specifically for propofol dosage taking into account physiological differences in specific populations. Their study highlights the advantages of fractional calculus in achieving robust performance with consistent dynamic responses despite the gain variations inherent in different body mass indexes [9]. Another fractional-order PI (FOPI) control strategy is designed to handle the BIS during both induction and maintenance phases; such an approach focuses on a personalised tuning method that enhances the system’s ability to reject disturbances caused by surgical stimuli, providing a stable hypnotic state compared to traditional integer-order alternatives [10].

Currently, alternative closed-loop control algorithms based on artificial intelligence (AI) are being actively investigated owing to their ability to process and analyse large volumes of data, including both patient-specific and population-level information. This capability enables the control system to be adapted either in a generalised manner or through personalised strategies, thereby optimising drug administration according to the specific surgical context. A further advantage of AI-based approaches is their capacity to integrate and process multiple variables of different natures, characterised by distinct scales and statistical distributions. In addition to anaesthesia monitoring parameters, physiological vital signs can also be incorporated as feedback signals. This heterogeneous combination of inputs not only facilitates the optimisation of anaesthetic management but also contributes to maintaining haemodynamic stability [11].

This work focuses on the design and evaluation of a variable set-point generation method for the control system proposed in [2], which is based on a PID-regulated general anaesthesia framework. Inspired by [12], the proposed method is based on the inversion of the PID control algorithm.

## 2. Methodology

### 2.1. Virtual Patient Model

The virtual patients used in this study are based on previously reported PK–PD compartmental models for propofol, remifentanil, and rocuronium [2,13,14,15]. These models are used only to simulate the patient response and to evaluate the proposed controller under reproducible conditions. They are not used by the ESynPID controller as an online model for state estimation or model-based inversion.

In the simulation framework, the drug infusion signals are applied to the continuous-time PK–PD virtual patient and numerically propagated using ode15s. The resulting monitor outputs, namely BIS, NOX, and NMB, are sampled and used as feedback signals for the discrete-time controller. Then, the controller operates on monitor signals and drug perfusion commands, whereas the PK–PD model is restricted to the virtual patient environment.

### 2.2. Enhanced Synergistic PID

The controller proposed in this work is a modification of synergistic multivariable PID (SynPID) which was introduced in our previous work [2]. The main concepts of SynPID are as follows:

1. OLP supervision:The anaesthetist’s clinical experience remains the for anaesthesia induction and maintenance and is incorporated into the controller through the Open-Loop Modulator.
2. Target feedforward: Setpoint feedforward is the effect of directly applying variations in the setpoint to the control action through proportional and derivative components.
3. Weighting: This is the mechanism by which setpoint feedforward influences the PID errors through modulation parameters (β and γ), resulting in a specific error for each branch of the PID. The modulation parameters are time variable and depend on the deviation between the measured value and the setpoint.
4. Unilateral control: This is only possible in the sense of administering medication to the human body; however, it is not possible to remove administered medication from it.
5. Multi-error: This refers to the fact that each PID has three distinct errors, one per PID branch, defined through weighting. Additionally, since there are three monitored variables, three errors arise per branch, resulting in a total of nine control errors.

The motivation for introducing the SynPID variant is the transition from a regulation-oriented control architecture to a tracking-oriented one. Regulation systems are typically designed around a constant reference input to maximise disturbance rejection, whereas tracking systems are formulated to ensure that the controlled variable follows dynamic changes in the reference trajectory. The SynPID operates under a fixed setpoint and exhibits intrinsic asymmetry. The Enhanced SynPID (ESynPID) is proposed to reformulate the error magnitude in two key respects:

1. When driven by a time-varying reference signal, the error magnitude can be deliberately amplified in either direction, thereby producing more pronounced increases or decreases in infusion rates compared with the original SynPID. These tracking systems aim to adjust the target according to the requirements imposed by surgical stimuli, based on variables derived from monitors and drug infusions, among the most relevant ones [12].
2. It adjusts the feedforward setpoint term (IsaPID concept) in such a manner that the controller exhibits symmetric actuation behaviour; i.e., it becomes equally assertive when reducing or administering medication. This symmetry enhances the controller’s robustness and improves the response performance to disturbances.

The ESynPID inherits from the SynPID the operational phases of induction, maintenance, and recovery, with the induction phase being the stage in which information is gathered for controller tuning upon its completion. It also inherits the concepts of OLP supervision, setpoint feedforward, weighting, unilateral control, and the multi-error controller. In addition, it incorporates the satellite modules for control to range and pump operation emulation.

**Figure 1:**
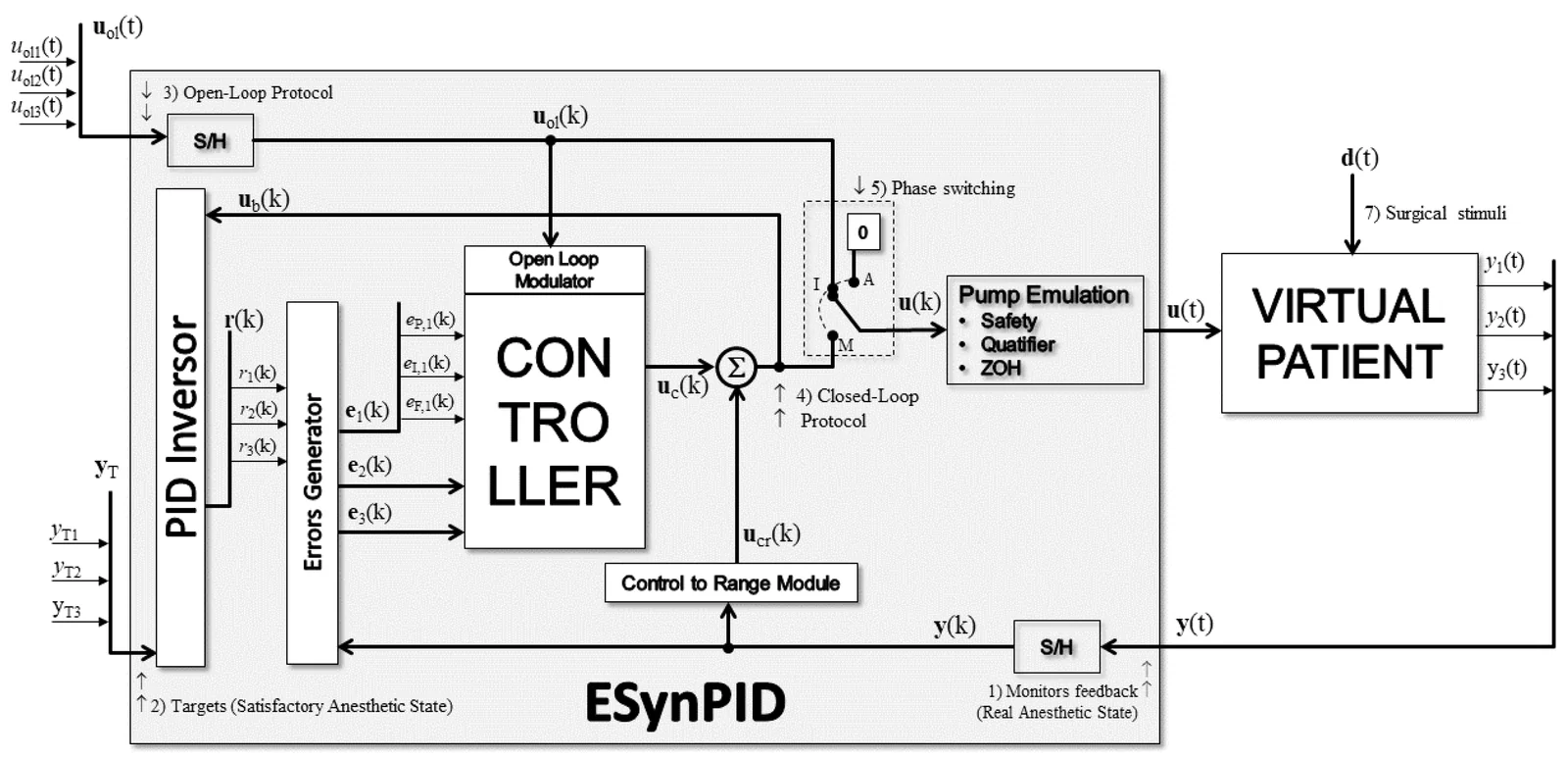
Enhanced SynPID block diagram.

The improvements of ESynPID with respect to its previous version are as follows (see Figure 1): (1) a new method to obtain the set-point feedforward weights, which condition the errors of each PID branch (modification of the block “Errors Generator”); (2) incorporation of an inverter block to obtain the variable reference signal (new “PID Inversor” block) and (3) upgrading the control-to-range module to define a symmetric behaviour.

The controller consists of five PIDs that relate the drug PPF to the BIS monitor ($c_{11}$), RMF-BIS ($c_{21}$), PPF-NOX ($c_{12}$), RMF-NOX ($c_{22}$), and RCN-NMB ($c_{33}$). The controllers $c_{13}$, $c_{23}$, $c_{31}$, and $c_{32}$ are not designed because there exists no documented relation between PPF or RMF and monitor NMB, nor between RCN with BIS or NOX. Each PID is defined with a different error in each branch, as follows:

(1) $$u_{i}\left(e_{j}\right)=K_{P,ij}\left(e_{P,j}\left(t\right)+\frac{1}{T_{I,ij}}\int_{0}^{t}e_{I,j}\left(\tau \right)\;d\tau +T_{D,ij}\frac{de_{F,j}\left(t\right)}{dt}\right),\;\left(i,j\right)\in S$$

with

S={(1,1),(1,2),(2,1),(2,2),(3,3)}.

and

$$i=\left\{\begin{array}{c} 1=\mathrm{PPF}\\ 2=\mathrm{RMF}\\ 3=\mathrm{RCN} \end{array}\right.,\;j=\left\{\begin{array}{c} 1=\mathrm{BIS}\\ 2=\mathrm{NOX}\\ 3=\mathrm{NMB} \end{array}\right.$$

where $K_{P,ij}$ is the proportional gain, $T_{I,ij}$ the integral action time, $T_{D,ij}$ the derivative action time, and $e_{j}\left(t\right)$ the deviation of monitor-*j* from the target value which defines the error in the Target Feedforward PID branch.

The errors of each branch of every PID are defined as a function of the reference signal ($r_{j}$), the controlled variable ($y_{j}$), and the weights ($\beta_{j}$, $\gamma_{j}$), according to the following:

(2) $$\begin{array}{ccc} e_{P,j}\left(t\right) & = & -\beta_{j}\left(t\right)r_{j}\left(t\right)+y_{j}\left(t\right),\\ e_{I,j}\left(t\right) & = & -r_{j}\left(t\right)+y_{j}\left(t\right),\\ e_{D,j}\left(t\right) & = & -\gamma_{j}\left(t\right)r_{j}\left(t\right)+y_{j}\left(t\right),\\ e_{F,j}\left(t\right) & = & e_{D,j}\left(t\right)-\frac{T_{D,ij}}{N}\cdot {\dot{e}}_{F,j}\left(t\right) \end{array}$$

where $e_{P,j}$ is the proportional error; $\beta_{j}$ is the proportional weighting; $r_{j}$ is the tracking set point; $y_{j}$ is the monitor; $e_{I,j}$ the integral error; $e_{D,j}$ the derivative error; $\gamma_{j}$ is the derivative weighting; $e_{F,j}$ is the derivative low-pass filtered error; $T_{D,ij}$ is the derivative action time; and *N* is the cut-frequency of the filter.

As illustrated in Figure 1, the Error Generator block receives two sequences, (1) the sampled monitor vector $y\left(k\right)={\left[y_{1}\left(k\right)\;y_{2}\left(k\right)\;y_{3}\left(k\right)\right]}^{⊤}$ and (2) the generated reference vector $r\left(k\right)={\left[r_{1}\left(k\right)\;r_{2}\left(k\right)\;r_{3}\left(k\right)\right]}^{⊤}$, to produce the three error vectors that feed the controller block:

(3) $$e_{1}\left(k\right)=\left[\begin{array}{c} e_{P,1}\left(k\right)\\ e_{I,1}\left(k\right)\\ e_{F,1}\left(k\right) \end{array}\right],\;e_{2}\left(k\right)=\left[\begin{array}{c} e_{P,2}\left(k\right)\\ e_{I,2}\left(k\right)\\ e_{F,2}\left(k\right) \end{array}\right],\;e_{3}\left(k\right)=\left[\begin{array}{c} e_{P,3}\left(k\right)\\ e_{I,3}\left(k\right)\\ e_{F,3}\left(k\right) \end{array}\right].$$

These discrete-time signals are obtained from the continuous-time weighted error definitions (2). Then, according to these errors, the corresponding discrete-time relations are

(4) $$\begin{array}{ccc} e_{P,j}\left(k\right) & = & -\beta_{j}\left(k\right)\;r_{j}\left(k\right)+y_{j}\left(k\right),\\ e_{I,j}\left(k\right) & = & -r_{j}\left(k\right)+y_{j}\left(k\right),\\ e_{D,j}\left(k\right) & = & -\gamma_{j}\left(k\right)\;r_{j}\left(k\right)+y_{j}\left(k\right), \end{array}$$

and, consistently with the implemented filtered derivative recursion,

(5) $$e_{F,j}\left(k\right)=p_{F,ij}\;e_{F,j}\left(k-1\right)+K_{F,ij}\;e_{D,j}\left(k\right),$$

where $0<p_{F,ij}<1$ and $K_{F,ij}>0$ are the discrete-time derivative-filter coefficients:

(6) $$\begin{array}{ccc} p_{F,ij} & = & e^{\frac{-T_{s}\cdot N}{T_{D,ij}}},\\ K_{F,ij} & = & 1-p_{F,ij}. \end{array}$$

Let the aggregate error vector be defined as

(7) $$e\left(k\right)={\left[\begin{array}{ccc} e_{1}^{⊤}\left(k\right) & e_{2}^{⊤}\left(k\right) & e_{3}^{⊤}\left(k\right) \end{array}\right]}^{⊤}\in R^{9}.$$

For each controller $c_{ij}$, the discrete-time SISO controller law can be written as

(8) $$u_{c,ij}\left(k\right)=K_{P,ij}e_{P,j}\left(k\right)+I_{ij}\left(k\right)+D_{ij}\left(k\right),$$

where $I_{ij}\left(k\right)$ is the integral state and $D_{ij}\left(k\right)$ is the derivative-filtered state.

The integral state updated through trapezoidal rule is defined by

(9) $$I_{ij}\left(k\right)={\mathrm{sat}}_{{\bar{I}}_{ij}}\left[I_{ij}\left(k-1\right)+\frac{K_{I,ij}T_{s}}{2}\left(e_{I,j}\left(k\right)+e_{I,j}\left(k-1\right)\right)\right],$$

The saturation operator in (9) models the anti-windup mechanism implemented, and guarantees that

(10) $$|I_{ij}\left(k\right)|\leq {\bar{I}}_{ij},\;\forall \;k.$$

The derivative-filtered state is defined by

(11) $$D_{ij}\left(k\right)=\left[p_{F,ij}D_{ij}\left(k-1\right)+\frac{K_{F,ij}K_{D,ij}}{T_{s}}\left(e_{D,j}\left(k\right)-e_{D,j}\left(k-1\right)\right)\right],$$

The control-to-range module generates auxiliary support perfusions $u_{cr,ij}\left(k\right)$ (see Section 2.7), and the resulting MISO contributions are combined inside the controller block. The total requested drug perfusion is then processed by the Pump Model Emulation, which impose non-negativity, quantisation and delivery limits. Hence, the applied infusion vector $u\left(k\right)={\left[u_{1}\left(k\right)\;u_{2}\left(k\right)\;u_{3}\left(k\right)\right]}^{⊤}$ satisfies

(12) $$0\leq u_{i}\left(k\right)\leq u_{Hi},\;i\in \left\{1,2,3\right\},$$

where $u_{Hi}$ is the maximum admissible infusion rate of the *i*-th pump.

The stability properties of the proposed discrete-time ESynPID controller, including the effects of the bounded weighting functions, anti-windup mechanism and pump emulation constraints, are formally analyzed in Appendix A.

### 2.3. Motivation: Range-Conditioned Tracking

**Figure 2:**
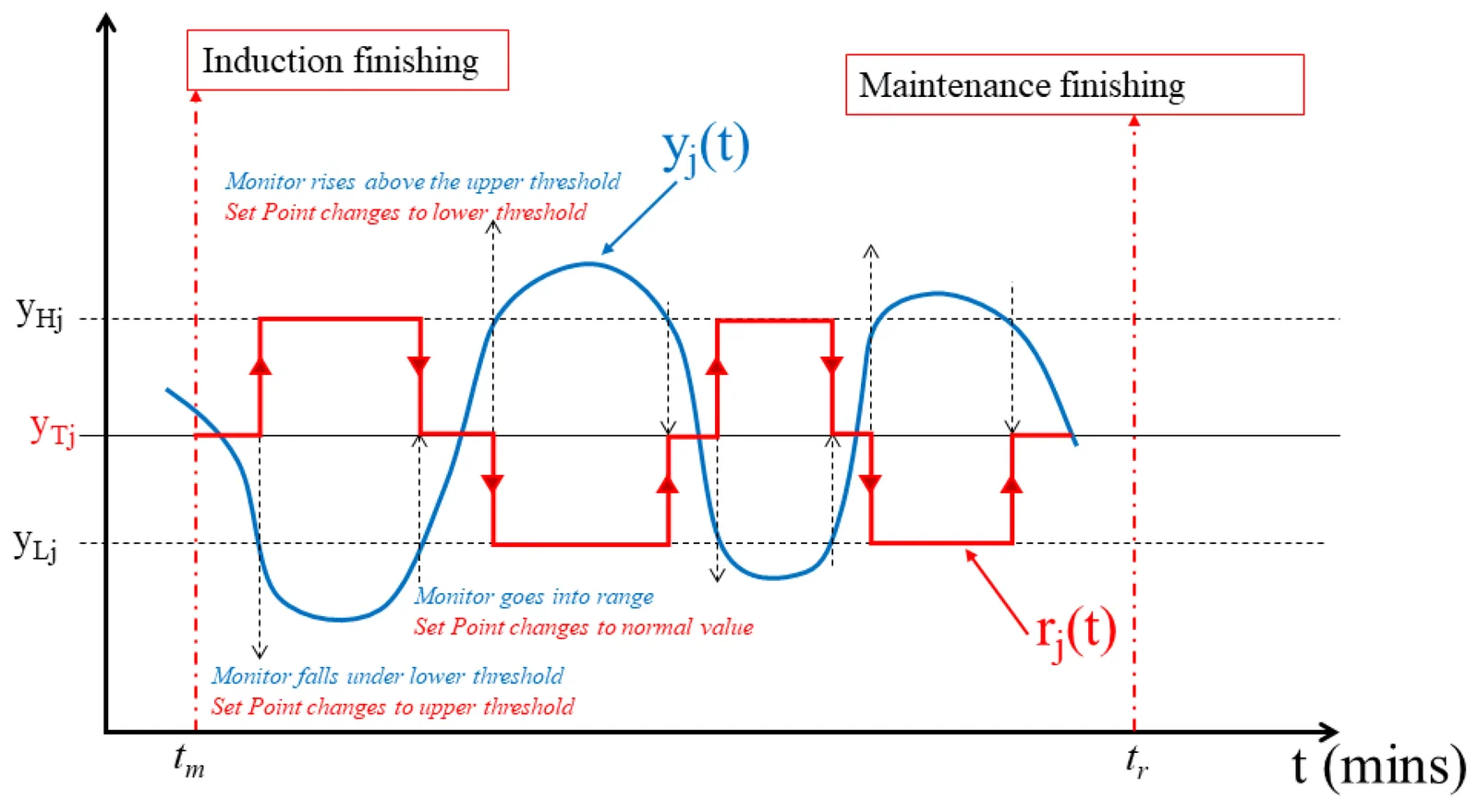
Setpoint variable conditioned by range thresholds.

The range-conditioned tracking method modifies the conventional PID error calculation when the controlled variable leaves the predefined safety range, see Figure 2. Instead of computing the error solely with respect to the target value, a threshold-conditioned reference is used to increase the error magnitude and therefore strengthen the control action. If the variable exceeds the upper limit, the error is calculated relative to the lower threshold; if it falls below the lower limit, it is calculated relative to the upper threshold. This approach enables a faster return of the controlled variable to the admissible range while preserving the standard error calculation when the variable remains within the safety limits.

In Equation (13), the computation of the range-conditioned tracking error is proposed by means of a piecewise-defined function:

(13) $$r_{j}\left(k\right)=\left\{\begin{array}{cc} y_{Lj}, & \mathrm{si}\;y_{j}\left(k\right)\geq y_{Hj}\\ y_{Tj}, & \mathrm{si}\;y_{Lj}<y_{j}\left(k\right)<y_{Hj}\\ y_{Hj}, & \mathrm{si}\;y_{j}\left(k\right)\leq y_{Lj} \end{array}\right.$$

where $r_{j}\left(k\right)$ is the variable reference signal (tracking); $y_{Lj}$ is the lower threshold of the range for the *j*-th monitor; $y_{Hj}$ is the upper threshold of the range for the *j*-th monitor; and $y_{Tj}$ is the fixed reference of the *j*-th monitor.

### 2.4. Target Feedforward PID Discretisation

Applying the discrete-time derivative and the trapezoidal integral to Equation (1), the z-transform of the control law is as follows:

(14) $$\begin{array}{cc} U_{i}\left(z\right)=\; & K_{P,ij}\left(-\bar{\beta_{j}}R_{j}\left(z\right)+Y_{j}\left(z\right)\right)\\ \; & +K_{I,ij}\frac{T}{2}\frac{1+z^{-1}}{1-z^{-1}}\left(-R_{j}\left(z\right)+Y_{j}\left(z\right)\right)\\ \; & +\frac{K_{D,ij}}{T}\left(1-z^{-1}\right)\left(-\bar{\gamma_{j}}R_{j}\left(z\right)+Y_{j}\left(z\right)\right)\\ =\; & \frac{K_{0,ij}+K_{1,ij}z^{-1}+K_{2,ij}z^{-2}}{1-z^{-1}}\;Y_{j}\left(z\right)\\ \; & -\frac{K_{3,ij}+K_{4,ij}z^{-1}+K_{5,ij}z^{-2}}{1-z^{-1}}\;R_{j}\left(z\right)\\ =\; & C_{ij}\left(z\right)\;Y_{j}\left(z\right)-B_{ij}\left(z\right)\;R_{j}\left(z\right),\;\left(i,j\right)\in S \end{array}$$

with *S* and i,j defined as in (2); where $U_{i}\left(z\right)$ denotes the z-transform of the control action associated with the *i*-th drug administration channel; $R_{j}\left(z\right)$ is the z-transform of the reference signal corresponding to the *j*-th physiological monitor; and $Y_{j}\left(z\right)$ represents the z-transform of the measured output of the *j*-th monitor.

The parameters $\bar{\beta_{j}}$ and $\bar{\gamma_{j}}$ are the mean setpoint feedforward weighting coefficients associated with the proportional and derivative actions, respectively, for the *j*-th monitor.

To simplify the Z-transform of Equation (1), it is assumed that the modulation parameters β and γ are small compared with the monitor values. Both parameters vary between 1 and their respective maximum values, B and G (see Section 2.6).

The terms $C_{ij}\left(z\right)$ and $B_{ij}\left(z\right)$ denote the discrete-time transfer functions associated with the feedback and feedforward paths, respectively, relating the *i*-th drug channel to the *j*-th monitored variable. In particular, if $\bar{\beta_{j}}=\bar{\gamma_{j}}=1$, then the feedforward and feedback dynamics become identical; i.e., $B_{ij}\left(z\right)=C_{ij}\left(z\right)$.

The coefficients $K_{n,ij}$, defined in Equation (15), are obtained from the PID tuning parameters and determine the discrete-time controller dynamics for each valid interaction pair (i,j)∈S.

Where $U_{i}\left(z\right)$ is the z-transform of control law of drug *i*-th; $R_{j}\left(z\right)$ is the z-transform of reference signal of monitor *j*-th; $Y_{j}\left(z\right)$ is the z-transform of monitor *j*-th; $\bar{\beta_{j}}$ and $\bar{\gamma_{j}}$ are the mean reference signal feedfoward weights of monitor *j*-th. The gains $K_{n,ij}$ shown in Equation (15) depend on the tuning of the PIDs in the following manner:

(15) $$\begin{array}{ccc} K_{0,ij} & = & K_{P,ij}+\frac{K_{I,ij}T_{s}}{2}+\frac{K_{D,ij}}{T_{s}}\\ K_{1,ij} & = & -K_{P,ij}+\frac{K_{I,ij}T_{s}}{2}-2\frac{K_{D,ij}}{T_{s}}\\ K_{2,ij} & = & \frac{K_{D,ij}}{T_{s}}\\ K_{3,ij} & = & \bar{\beta_{j}}K_{P,ij}+\frac{K_{I,ij}T_{s}}{2}+\bar{\gamma_{j}}\frac{K_{D,ij}}{T_{s}}\\ K_{4,ij} & = & -\bar{\beta_{j}}K_{P,ij}+\frac{K_{I,ij}T_{s}}{2}-2\bar{\gamma_{j}}\frac{K_{D,ij}}{T_{s}}\\ K_{5,ij} & = & \bar{\gamma_{j}}\frac{K_{D,ij}}{T_{s}}, \end{array}$$

where (i,j)∈S is as defined in (2); $K_{P,ij}$, $K_{I,ij}$, and $K_{D,ij}$ denote the proportional, integral, and derivative gains, respectively, associated with the interaction between the *i*-th drug infusion channel and the *j*-th physiological monitor; and $T_{s}$ denotes the discrete-time sampling period. $\bar{\beta_{j}}$ is the mean proportional feedforward weight and $\bar{\gamma_{j}}$ is the mean derivative feedforward weight.

### 2.5. Discrete Target Feedforward PID Inversion

For the direct effect–action pathways (BIS–PPF, NOX–RMF and NMB–RCN), the condition i=j is imposed. Therefore, only the diagonal controllers are considered and the double subscript notation is simplified as $C_{ii}\left(z\right)\equiv C_{i}\left(z\right)$, $B_{ii}\left(z\right)\equiv B_{i}\left(z\right)$ and $K_{m,ii}\equiv K_{mi}$, for m=0,…,5. Under this notation, solving (14) for the reference signal transform $R_{i}\left(z\right)$ and applying the inverse *z*-transform yields the recursive reference-generation equation:

(16) $$\begin{array}{cc} R_{i}\left(z\right)= & \frac{C_{i}}{B_{i}}\cdot Y_{i}\left(z\right)-\frac{1}{B_{i}}\cdot U_{i}\left(z\right)\\ R_{i}\left(z\right)= & \frac{\left(K_{0i}+K_{1i}z^{-1}+K_{2i}z^{-2}\right)Y_{i}\left(z\right)-\left(1-z^{-1}\right)U_{i}\left(z\right)}{K_{3i}+K_{4i}z^{-1}+K_{5i}z^{-2}}\\ r_{i}\left(k\right)= & -\frac{K_{4i}}{K_{3i}}r_{i}\left(k-1\right)-\frac{K_{5i}}{K_{3i}}r_{i}\left(k-2\right)+\frac{K_{0i}}{K_{3i}}y_{i}\left(k\right)\\ \; & +\frac{K_{1i}}{K_{3i}}y_{i}\left(k-1\right)+\frac{K_{2i}}{K_{3i}}y_{i}\left(k-2\right)-\frac{1}{K_{3i}}\left(u_{i}\left(k\right)-u_{i}\left(k-1\right)\right) \end{array}$$

where $u_{i}\left(k\right)$ denotes the control action associated with the *i*-th drug administration channel and $U_{i}\left(z\right)$ its *z*-transform; $r_{i}\left(k\right)$ is the reference signal associated with the *i*-th direct monitor–drug pathway and $R_{i}\left(z\right)$ its corresponding *z*-transform; and $y_{i}\left(k\right)$ is the measured output of the same pathway with $Y_{i}\left(z\right)$ its *z*-transform. Then, once the discrete PID gains $K_{0i},\ldots ,K_{5i}$ are known, together with the previous reference samples, the measured outputs, and the increment of the drug infusion rate, the recursive reference-generation law given by (16) is completely determined.

Under steady-state conditions, several implications arise from the presence of the integral action in the PIDs controllers. For constant reference signals and stable closed-loop operation, the tracking error converges to zero. Then, the proportional and derivative contributions vanish, whereas the integral contribution converges to a constant value.

Therefore, considering steady-state conditions in (16), the signals satisfy

(17) $$r_{i}\left(k\right)=r_{i}\left(k-1\right)=r_{i}\left(k-2\right)=r_{i}^{★},$$

(18) $$y_{i}\left(k\right)=y_{i}\left(k-1\right)=y_{i}\left(k-2\right)=y_{i}^{★},$$

and

(19) $$u_{i}\left(k\right)=u_{i}\left(k-1\right)=u_{i}^{★},$$

which implies

(20) $$u_{i}\left(k\right)-u_{i}\left(k-1\right)=0.$$

and $\beta_{i}=\gamma_{i}=1$ (see Section 2.6) due to $y_{i}^{★}=r_{i}^{★}$ (condition for null error).

Substituting these conditions into (16) yields

(21) $$\left(K_{3i}+K_{4i}+K_{5i}\right)r_{i}^{★}=\left(K_{0i}+K_{1i}+K_{2i}\right)y_{i}^{★}.$$

For the direct pathways the PIDs coefficients satisfy

(22) $$K_{0i}+K_{1i}+K_{2i}=K_{3i}+K_{4i}+K_{5i},$$

(23) $$r_{i}^{★}=y_{i}^{★}.$$

Hence, under steady-state conditions, the generated reference converges to the measured physiological variable. This result guarantees that the reference-generation mechanism does not introduce any steady-state offset with respect to the monitored output.

The steady-state analysis presented above reveals an important limitation of the inversion law (16). Since the generated reference satisfies $r_{i}^{★}=y_{i}^{★}$, the reference signal eventually converges to the measured monitor output. Hence, the generated reference loses its driving role and behaves as a replica of the controlled variable rather than as a setpoint capable of steering the physiological monitor towards a desired target.

To recover the setpoint-driving capability, the feedback term associated with the monitor output is replaced by a constant target value $y_{Ti}$. In this way, the inversion law is no longer referenced to the current monitor value but to the desired operating point.

A second practical consideration concerns the signal used as input to the inverter. Since the objective of the inversion mechanism is to estimate the reference required to produce a given drug infusion, the inverter must receive the actual perfusion delivered to the patient. Therefore, the input of the inverter is defined as the feedback perfusion $u_{bi}\left(k\right)$, obtained from the sum of the PID contribution and the support action provided by the Range–Control module. Both quantities are evaluated at the previous sampling instant, k−1, to avoid an algebraic loop:

(24) $$\begin{array}{ccc} u_{bi}\left(k\right) & = & \sum_{j=1}^{2}\left(u_{c,ij}\left(k-1\right)+u_{cr,ij}\left(k-1\right)\right),\\ u_{b3}\left(k\right) & = & u_{c33}\left(k-1\right)+u_{cr,33}\left(k-1\right), \end{array}$$

where $u_{c,ij}\left(k\right)$ is the control action generated by the PID controller $c_{ij}$ and $u_{cr,ij}\left(k\right)$ is the auxiliary perfusion supplied by the Range–Control module.

Finally, in order to regulate the influence of the feedback perfusion on the inversion process, an inversion gain $K_{ui}$ is introduced. This parameter allows the contribution of the perfusion increment to be adjusted, thereby providing an additional degree of freedom for tuning the inversion dynamics without compromising closed-loop stability.

Taking the above considerations into account, the final recursive inversion law used to generate the variable setpoint signal is given by

(25) $$r_{i}\left(k\right)=-\frac{K_{4i}}{K_{3i}}\;r_{i}\left(k-1\right)-\frac{K_{5i}}{K_{3i}}\;r_{i}\left(k-2\right)+\frac{K_{0i}+K_{1i}+K_{2i}}{K_{3i}}\;y_{Ti}-\frac{K_{ui}}{K_{3i}}\;\Delta u_{bi}\left(k\right)$$

where $r_{i}\left(k\right)$ is the generated setpoint associated with the *i*-th monitor, $y_{Ti}$ is the desired target value of the corresponding monitor, $u_{bi}\left(k\right)$ is the feedback perfusion defined in (24), and $K_{ui}$ is the inversion gain.

### 2.6. Weighting

In this section, the setpoint feedforward weights of the PIDs are defined. The weights $\beta_{j}\left(k\right)$ and $\gamma_{j}\left(k\right)$ involved (through the mean values) in the calculation of the gains presented in Equation (15) determine the controller’s behaviour, with two possible modes: asymmetric and symmetric. The asymmetric mode was presented in our previous work [2], whereas in this work we propose a symmetric operating mode, for which the following criteria are used:

1. If the monitor is equal to the target, the weights take a unit value ($\beta_{j}=\gamma_{j}=1$). Only in this case does the PID with setpoint feedforward behave as a standard PID.
2. The weights are always positive and their minimum value is 1.
3. To ensure stability, the value of the weights cannot exceed a maximum, which is reached when the monitor exceeds the out-of-range threshold.
4. Absolute linear growth between 1 and the maximum value.

Given the stated criteria, and understanding symmetry as the condition in which the values of the weights coincide when the monitor values deviate equally from the target both above and below it, Equation (26) defines the weight $\beta_{j}\left(k\right)$, and Equation (27) defines the weight $\gamma_{j}\left(k\right)$.

(26) $$\beta_{j}\left(k,y_{j}\right)=\left\{\begin{array}{cc} \frac{\left(B-1\right)|y_{j}\left(k\right)-Y_{Tj}|}{Y_{Bj}-Y_{Tj}}+1, & |y_{j}\left(k\right)-Y_{Tj}|\leq Y_{Bj}-Y_{Tj},\\ B, & \mathrm{otherwise} \end{array}\right.$$

(27) $$\gamma_{j}\left(k,y_{j}\right)=\left\{\begin{array}{cc} \frac{\left(G-1\right)|y_{j}\left(k\right)-Y_{Tj}|}{Y_{Gj}-Y_{Tj}}+1, & |y_{j}\left(k\right)-Y_{Tj}|\leq Y_{Gj}-Y_{Tj},\\ G, & \mathrm{otherwise} \end{array}\right.$$

where for the monitor *j*-th, $y_{j}$ is its value; $Y_{Tj}$ is its target; *B* and *G* are the maximum weight values for $\beta_{j}$ and $\gamma_{j}$ respectively; and $Y_{Bj}$ and $Y_{Gj}$ are the symmetric thresholds out of range.

### 2.7. Control-to-Range Module Upgrade

The control-to-range module (CTRM) has a slope determined by the weight $\beta_{j}\left(k\right)$; consequently, the concept of symmetry alters the behaviour of this module with respect to the definition of asymmetry. To ensure consistency with the symmetric approach, the module is updated to exhibit the same behaviour when the monitored variables fall below the lower threshold as when they exceed the upper threshold (the asymmetric behaviour was defined in [2]).

The CTRM perfusion ($u_{cr,i}$) is computed as follows:

(28) $$u_{cr,i}\left(k\right)=\left\{\begin{array}{cc} K_{Pi}\cdot \left(y_{i}\left(k\right)-B\cdot y_{Li}\right), & y_{Lj}\geq y_{i}\left(k\right),\\ K_{Pi}\cdot \left(y_{i}\left(k\right)-y_{Li}\left(B-1\right)-y_{Hi}\right), & y_{Hi}\leq y_{i}\left(k\right),\\ 0, & \mathrm{otherwise} \end{array}\right.$$

where $y_{Hi}$ is the upper threshold of the *i*-th monitor and $y_{Li}$ is the lower threshold of the *i*-th monitor.

### 2.8. Inversion Gain Tuning

The inversion gain ($K_{ui}$) was introduced for stability reasons, with the aim of reducing oscillations and increasing relative stability. This gain is tuned heuristically by pursuing two main objectives:

1. To maintain the reference signal within a relative range of ±30% of the BIS target value (range 25–45), ±50% of the NOX target value (range 15–45), and ±100% of the NMB target value (range 0–20).
2. To decouple the feedback term of the perfusion from the inverter and the weighting (see Equation (25)).

With these criteria, the inversion gain is personalised for each monitor and each patient after the PID gains have been computed. The gain is defined as

(29) $$K_{ui}=\alpha_{i}\frac{K_{3i}}{K_{0i}},$$

where $\alpha_{i}$ is a dimensionless heuristic coefficient, $K_{3i}$ is the coefficient associated with the setpoint-feedforward part of the discretised PID, and $K_{0i}$ is the coefficient associated with the feedback part of the discretised PID, as defined in (15).

The tuning of $K_{ui}$ is not performed as an online optimisation process, but as a patient- and monitor-dependent heuristic adjustment applied after the PID gains have been obtained. The procedure consists of the following steps. First, the PID parameters are fixed according to the tuning criterion reported in Table 1. Then, the coefficients $K_{0i}$ and $K_{3i}$ are computed from (15). Finally, the coefficient $\alpha_{i}$ is selected for each monitor to regulate the effect of the feedback-perfusion increment in the inversion law (25).

The reason for defining $K_{ui}$ as in (29) is that the perfusion-feedback contribution in (25) becomes

(30) $$\frac{K_{ui}}{K_{3i}}\Delta u_{bi}\left(k\right)=\frac{\alpha_{i}}{K_{0i}}\Delta u_{bi}\left(k\right).$$

Then, the direct influence of the feedback perfusion on the generated setpoint is mainly regulated by $\alpha_{i}$, while the explicit dependence on the setpoint-feedforward coefficient $K_{3i}$ is cancelled. This normalisation is introduced to reduce excessive sensitivity of the PID Inversor to variations in the weighting terms $\beta_{i}$ and $\gamma_{i}$.

In this work, $\alpha_{i}$ is selected by checking two practical conditions: (1) the generated reference must remain inside the admissible relative range defined for each monitor; (2) the feedback-perfusion term must not induce large oscillations in the generated setpoint or in the drug-infusion profile. Small values of $\alpha_{i}$ produce a weak inversion effect and make the generated reference closer to a constant target, whereas large values increase the tracking activity but may amplify oscillations. Therefore, the selected values represent a compromise between tracking capability and relative closed-loop stability. The values used in the simulations are reported in Table 2.

**Table 1:** PID tuning (MU: monitorisation units).

VP No.	$K_{p}$ ($\times 10^{-3}$)			$T_{i}$ (s)			$T_{d}$ (s)		
	$c_{11}$ (mg/s/MU)	$c_{22}$ (µg/s/MU)	$c_{33}$ (µg/s/MU)	$c_{11}$	$c_{22}$	$c_{33}$	$c_{11}$	$c_{22}$	$c_{33}$
1	0.53	0.24	17.27	36.73	39.86	44.38	13.90	16.87	12.54
2	0.22	0.26	9.71	47.85	41.22	61.83	26.50	16.56	19.58
3	0.47	0.22	10.84	36.73	39.86	56.61	13.90	16.87	17.13
4	0.53	0.22	18.06	29.81	32.64	38.13	11.75	14.83	10.28
5	0.23	0.32	13.43	30.98	36.64	42.27	11.51	17.67	12.88
6	0.71	0.20	14.90	27.92	38.35	43.00	8.63	17.23	12.75
7	0.40	0.38	18.86	27.11	32.64	36.87	8.77	14.83	10.47
8	0.18	0.38	9.02	36.64	32.64	48.27	17.67	14.83	15.20
9	1.85	0.38	8.06	24.19	36.73	61.83	5.98	13.90	19.58
10	1.40	0.54	11.94	25.59	42.46	50.53	5.80	16.30	14.82
11	0.37	0.37	19.86	26.21	32.64	36.87	8.93	14.83	10.47
12	0.37	0.41	23.84	32.04	38.35	39.27	11.30	17.23	10.11

**Table 2:** Typical ESynPID simulation parameters for each monitor: Constant targets, symmetric ranges, and inversion-gain heuristic coefficients.

Parameter	Monitor		
	BIS	NOX	NMB
*i*-th monitor	1	2	3
$y_{Ti}\pm Y_{Hi}$	50±10	30±10	10±5
$\alpha_{i}$	0.04	0.02	0.04

### 2.9. Retroactive PID State Alignment

After the induction phase, two very important events occur in the application of ESynPID: (1) immediately upon completion of the induction phase, the five PIDs are tuned using optimal tuning mechanisms, specifically based on the IAE criterion; (2) the switching from OLP to CLP with ESynPID is performed. It is during this transition that unexpected transients arise, mainly due to the initial execution of the algorithms with variables not properly initialised, default values being applied, zero infusion rates, constant and unitary weighting, correction modules with zero outputs, and uninitialised integral terms, among other key factors.

**Figure 3:**
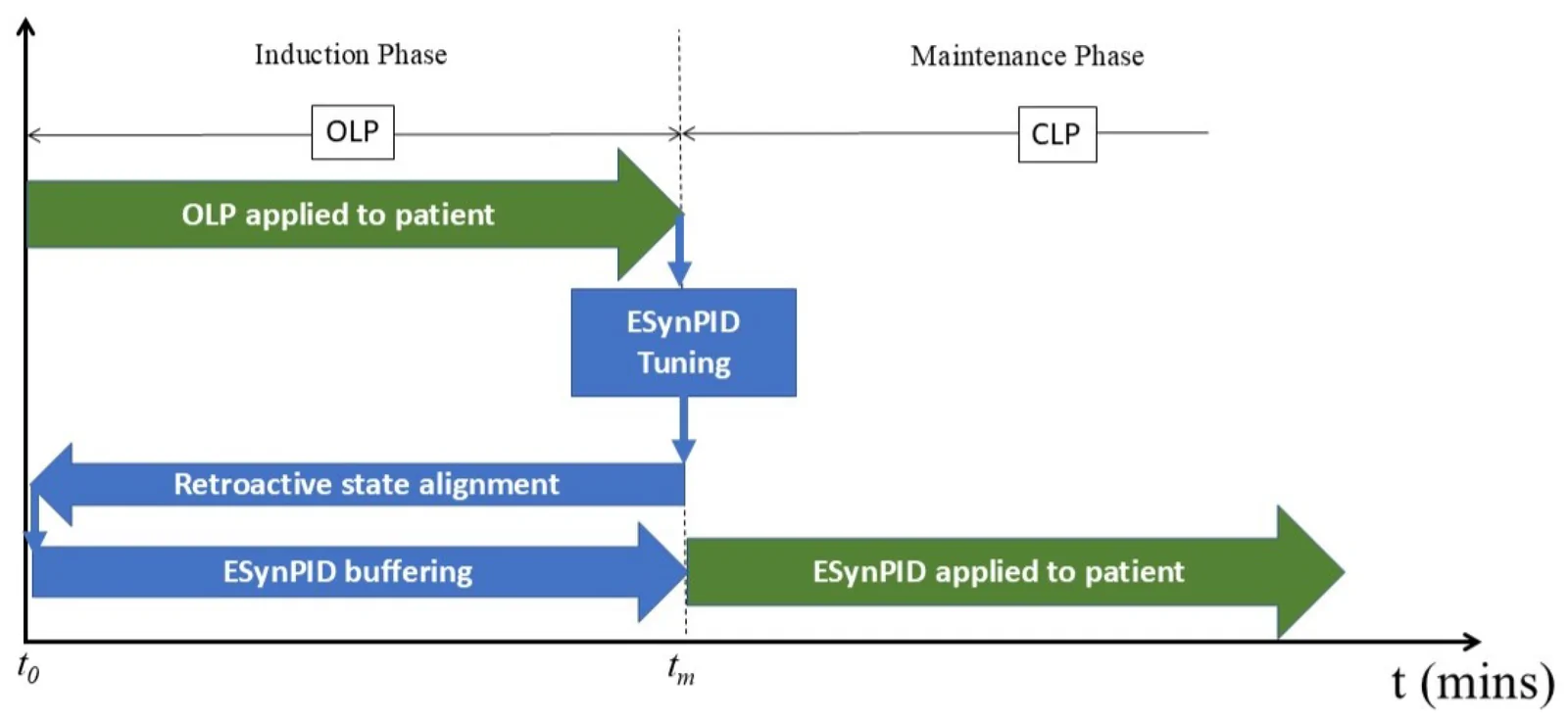
Retroactive PID state alignment procedure.

To mitigate the effect of this transient, a backward-execution technique is employed once the PIDs have been tuned [16,17] (see Figure 3 for details). The process returns to a virtual starting point of the induction phase (with the patient already induced and prepared for surgery), and the ESynPID is fed with the monitor values obtained under OLP. This prepares the controller to propose the phantom infusion rates of the drugs, as these cannot actually be applied to the patient. All internal PID variables then take values different from their initialisation, and, by the time the system returns to the present moment (i.e., the switching from OLP to CLP), the internal variables follow dynamics consistent with the monitored signals, which, in the subsequent instant, are already governed by the PID control laws.

### 2.10. Performance Evaluation

The performance evaluation includes the monitors, perfusions, and other features such as histograms, minimum monitor values, and time to target [2].

The monitor metrics are computed with respect to the fixed clinical target $y_{Tj}$ of the *j*-th monitor, and not with respect to the time-varying setpoint $r_{j}\left(k\right)$ generated by the ESynPID target generator. The variable setpoint $r_{j}\left(k\right)$ is used only internally by the controller to modify the control action, whereas $y_{Tj}$ is used as the common reference for performance evaluation across SynPID and ESynPID configurations.

The monitor metrics are: Median Performance Error (MDPE) describes how the monitors are distributed above or below the fixed target $y_{Tj}$ (see Section 2.3 for details); global score (GS) summarises the performance of the control; NADIR measures the minimum observed monitor value; higher range (HRG) is the time during which the monitors rise above the upper threshold; range (RG) is the time during which the monitors remain within the ideal range; and lower range (LRG) is the time during which the monitors drop below the lower threshold. GS, NADIR, and RG are considered the primary metrics, as they provide a sufficiently accurate assessment of performance.

The perfusion metrics considered are: (1) Total Variation (TV), which quantifies the variability in the perfusion rate required to maintain the patient within the target range, and (2) the total amount of drug administered during the maintenance phase.

### 2.11. Experimental Design

The proposed experiments aim to compare the performance of the SynPID strategy introduced in our previous work [2] with the enhanced ESynPID approach presented in this study. The formulation in [2] employed asymmetric weighting, an asymmetric Range–Control module, and a regulation scheme based on a constant target. In contrast, the proposed ESynPID incorporates symmetric weighting with B=G=3 (see Equations (26) and (27)), a symmetric Range–Control module (see Equation (28) and Table 2), and a tracking-oriented target-generation scheme (see Equations (25) and (29) and Table 2).

To ensure a fair comparison, both approaches (ESynPID and SynPID) use the same PID tuning procedure established and discussed in our previous work [2]. The PID gains are reproduced in Table 1 because they are required to determine the inverter gains shown in Equation (15), which directly depend on the PID controller gains. Both approaches use the same controller matrix structure and pump emulation mechanism. The virtual patients, manual drug administration protocols, surgical scenarios, time distributions across the anaesthesia phases, surgical stimuli, sampling period, and integration mechanism—among other key aspects—are those defined in our previous work, in which the performance of the SynPID was evaluated [2].

It should be noted that the surgical stimulus modeled in this study (Struy’s stimuli [18]) specifically affects the hypnotic (BIS) and nociceptive (NOX) indicators. In contrast, the neuromuscular blockade (NMB) monitor is not subject to external surgical disturbances. Consequently, once the controller drives the NMB indicator to the desired target setpoint, no continuous adjustments to the rocuronium infusion rate are triggered by external perturbations.

#### 2.11.1. Experiment 1: SynPID vs. ESynPID Performance

This experiment evaluates the overall performance obtained when the tracking mechanism and the symmetric weighting strategy are incorporated into the original SynPID formulation. The comparison is carried out over the cohort of 12 virtual patients under the Struys surgical stimulus profile. The objective is to assess whether the modifications introduced in ESynPID improve the robustness of the control law, even if this improvement may lead to larger oscillations in drug perfusions due to the tracking-oriented target generation.

#### 2.11.2. Experiment 2: Weighting Effect: Asymmetric vs. Symmetric

This experiment evaluates the partial performance effect of modifying only the weighting strategy, from asymmetric to symmetric, while keeping the same tracking-oriented target-generation scheme. Thus, the asymmetric tracking configuration is compared with the symmetric ESynPID configuration. The objective is to isolate the effect of the weighting strategy on drug perfusions when the setpoint is generated through the tracking mechanism.

#### 2.11.3. Experiment 3: Setpoint Effect: Regulator vs. Tracking

This experiment evaluates the partial performance effect of introducing the tracking-oriented target-generation scheme while keeping the weighting strategy unchanged. Specifically, the asymmetric regulator configuration is compared with the asymmetric tracking configuration. The objective is to isolate the effect of the tracking mechanism on drug perfusions when the weighting strategy remains asymmetric.

## 3. Results

The results obtained for each experiment are presented below. The Table 3 summarises the inversion gain extracted after applying the proposed methodology to the cohort of 12 virtual patients, according to the experimental design.

For every experiment shown in Table 4, Table 5, Table 6, Table 7, Table 8 and Table 9, each column reports the performance metrics defined in Section 2.10 for evaluating the results of the different experiments. For each metric, the corresponding values of the BIS and NOX monitors are shown. The rows represent the application of the different protocols, specifying the operating mode, the weighting strategy, and a pseudonym used to identify each dataset:

1. **Range** corresponds to the application of SynPID with a setpoint defined by (13), considering both the asymmetric weighting strategy reported in [2] and the symmetric weighting strategy defined in (26) and (27). This case provides the performance obtained with a tracking signal that depends on the monitor ranges and monitor values.
2. **Regulator** corresponds to the application of the original SynPID strategy [2].
3. **Tracking** corresponds to the proposed ESynPID strategy.

**Table 3:** Average inversion gain.

VP No.	$K_{u}$ ($\times 10^{-3}$ Unitless)		
	B	$c_{22}$	$c_{33}$
1	125.55	61.64	42.66
2	122.50	54.97	46.27
3	114.30	55.37	45.26
4	118.80	66.82	42.30
5	119.43	59.11	43.91
6	109.66	60.71	42.76
7	112.61	64.22	47.06
8	113.63	50.12	44.18
9	112.85	72.76	47.22
10	103.34	57.16	43.71
11	112.69	54.91	44.03
12	121.32	64.33	42.95

### 3.1. Experiment 1: SynPID vs. ESynPID

Table 4 presents the performance evaluation of the proposed ESynPID strategy with respect to the original SynPID configuration, based on the median values obtained across a cohort of 12 virtual patients. Table 5 reports the primary performance metrics (see Section 2.10) as mean values and standard deviations. In this comparison, the asymmetric regulator mode is considered as the baseline SynPID case, whereas the symmetric tracking mode corresponds to the proposed ESynPID approach.

- **MDPE**: For BIS, the monitor remains below the target value of 50 MU in both cases. However, ESynPID (Tracking) reduces the deviation from −14 MU to −10 MU, placing the BIS response 4 MU closer to the target, which corresponds to the 40.0% improvement reported in the table. For NOX, the monitor remains above the target value of 30 MU, but the deviation increases from 9.5 MU to 18.75 MU. This means that the NOX response moves 9.25 MU farther from the target, corresponding to a deterioration of 49.3%.
- **GS**: The global score indicates that ESynPID (Tracking) improves the BIS performance by 17.1%, decreasing from 31.2 to 26.6 score units. In contrast, the NOX global score worsens slightly by 5.3%, increasing from 57.9 to 61.1 score units. Nevertheless, when compared with the symmetric range-based tracking case, the inversion-based tracking strategy improves the GS metric for both BIS and NOX.
  For ESynPID (Tracking), the mean BIS value is close to the median and 18.8% lower and a deviation 18.6% greater than SynPID (Regulator). However, the mean NOX value is 13.7 higher than the median, indicating that the distribution is skewed toward higher values, also is 3.2% lower than SynPID (Regulator). In addition, the NOX standard deviation is relatively large (57.5), suggesting that at least one virtual patient is poorly controlled under this configuration, but is 28.5% lower than SynPID (Regulator).
- **NADIR**: For BIS, ESynPID (Tracking) reduces the NADIR metric from 21.4 to 18.3 score units, corresponding to a reduction of 16.5%. For NOX, the change is negligible, since the metric remains practically unchanged around 8.2 score units, with a variation of only 0.2%.
  In general, the mean BIS NADIR is 11 MU and mean NOX NADIR is 7.3 MU, both higher than the median under ESynPID (Tracking), no relative differences exist respect to SynPID (Regulator). This indicates that the distributions are shifted toward higher monitor values, although the dispersion remains acceptable, with standard deviations of 5.4 MU (25.9%) for BIS and 4.3 MU (2.4%) for NOX.
- **HRG**: ESynPID (Tracking) reduces the percentage of samples above the upper range for both monitors. For BIS, HRG decreases from 22.6 to 19.3 score units, corresponding to a reduction of 17.1%. For NOX, the reduction is much smaller, from 6.1 to 6.0 score units, equivalent to 1.7%.
- **RG**: The time within the desired range increases for BIS, from 61.0 to 66.2 score units, which represents an improvement of 7.9%. For NOX, RG decreases slightly from 62.2 to 61.8 score units, corresponding to a minor reduction of 0.6%. Compared with the symmetric range-based tracking case, the inversion-based tracking strategy increases the time in range for both BIS and NOX. Regarding the time in range, the mean and median values are similar, suggesting approximately symmetric distributions. The dispersion is acceptable for both monitors, although it is slightly larger for NOX.
- **LRG**: For BIS, ESynPID (Tracking) reduces the percentage of samples below the lower range from 16.4 to 14.5 score units, corresponding to an improvement of 13.1%. For NOX, LRG increases slightly from 31.7 to 32.2 score units, producing a small deterioration of 1.6%.

**Table 4:** Monitor metrics in medians for Experiment 1, ESynPID (Tracking) vs. SynPID (Regulator [2]).

Exp.1		MDPE		GS		NADIR		HRG		RG		LRG	
Weighting	Mode	BIS	NOX	BIS	NOX	BIS	NOX	BIS	NOX	BIS	NOX	BIS	NOX
Symmetric	Range	−13.0	16.5	28.9	76.2	16.8	6.8	20.7	6.0	62.9	59.2	16.5	34.8
Asymmetric	Regulator	−14.0	9.5	31.2	57.9	21.4	8.2	22.6	6.1	61.0	62.2	16.4	31.7
Symmetric	Tracking	−10.0	18.8	26.6	61.1	18.3	8.2	19.3	6.0	66.2	61.8	14.5	32.2
ESynPID vs. SynPID		−40.0%	49.3%	−17.1%	5.3%	−16.5%	0.2%	−17.1%	−1.7%	7.9%	−0.6%	−13.1%	1.6%

**Table 5:** Primary metrics in mean (SD) for Experiment 1, ESynPID (Tracking) vs. SynPID (Regulator [2]).

Exp.1		GS		NADIR		RG	
Weighting	Mode	BIS	NOX	BIS	NOX	BIS	NOX
Symmetric	Range	31.3 (7.6)	87.4 (55.8)	29.3 (4.3)	15.7 (4.3)	55.6 (16.9)	59.2 (12.7)
Asymmetric	Regulator	32.8 (5.7)	77.2 (73.9)	29.1 (4.0)	15.2 (4.2)	60.9 (4.8)	62.2 (12.7)
Symmetric	Tracking	27.6 (7.0)	74.8 (57.5)	29.3 (5.4)	15.5 (4.3)	66.2 (4.6)	61.8 (12.1)

### 3.2. Experiment 2: Weighting Effect Under Tracking: Asymmetric vs. Symmetric

Table 6 presents the performance evaluation of the tracking scheme when only the weighting strategy is modified, based on the median values obtained across a cohort of 12 virtual patients. Table 7 reports the primary performance metrics (see Section 2.10) as mean values and standard deviations. In this experiment, the asymmetric tracking configuration is compared with the symmetric tracking configuration, which corresponds to the proposed ESynPID setting. Therefore, the objective is to isolate the effect of the weighting strategy on the performance metrics and on the drug perfusion behaviour.

- **MDPE**: For BIS, both configurations remain below the target value of 50 MU. However, the symmetric tracking strategy reduces the deviation from −13 MU to −10 MU, placing the BIS response 3 MU closer to the target, which corresponds to the 30.0% improvement reported in the table. For NOX, the monitor remains above the target value of 30 MU; nevertheless, the deviation increases from 12.75 MU to 18.75 MU, meaning that the response moves 6 MU farther from the target, with a deterioration of 32.0%.
- **GS**: The global score shows that the symmetric weighting strategy improves the BIS performance by 11.3%, decreasing from 29.6 to 26.6. In contrast, the NOX global score worsens by 11.2%, increasing from 54.2 to 61.1. When compared with the range-based configurations, the inversion-based tracking scheme improves the NOX metric for both weighting strategies, while for BIS the improvement is clearly observed under the symmetric weighting configuration. For ESynPID, corresponding to the symmetric tracking configuration, the mean BIS value is close to the median, with a standard deviation of 7.3. Although the mean BIS value is 12.7% lower than that obtained with the asymmetric range configuration, no substantial difference is observed with respect to that case. For NOX, the mean value under symmetric tracking is 13.7 MU higher than the median, indicating a distribution skewed toward higher values. However, this mean value is 4.0% lower than that obtained with the asymmetric tracking configuration. The relatively large NOX standard deviation (60.2 MU) suggests that at least one virtual patient remains poorly controlled. Although the NOX dispersion is 28.6% lower than in the asymmetric tracking case, changing the weighting strategy alone does not fully resolve this behaviour. An additional observation is that, for NOX, the median GS under asymmetric tracking is 6.9 MU lower than under ESynPID, whereas the mean GS is reduced by only about 3 units. This difference between median- and mean-based interpretations suggests that the NOX response is strongly affected by inter-patient variability.
- **NADIR**: The symmetric tracking strategy increases the NADIR metric for both monitors. For BIS, the value increases from 16.4 to 18.3 score units, while for NOX it increases from 7.3 to 8.2 score units, corresponding to variations close to 10.8% and 11.0%, respectively. Compared with the range-based configuration, the tracking scheme provides a more favourable NADIR behaviour when the symmetric weighting strategy is used.
  In general, the mean BIS NADIR is 11 MU higher than the median under ESynPID (Tracking), whereas for NOX the difference is 7.3 MU, For the case of NADIR comparing BIS vs. NOX there is not significative difference due the values are practically similar. This indicates that the distributions are shifted toward higher monitor values, with acceptable dispersion values of 5.6 MU for BIS and 4.5 MU for NOX. Changing the weighting strategy to the symmetric mode produces only a weak improvement in NADIR, since the metric increases slightly for both BIS and NOX.
- **HRG**: The percentage of samples above the upper range is slightly reduced for BIS, decreasing from 20.2 to 19.3, which corresponds to a 4.7% improvement. For NOX, HRG increases from 5.7 to 6.0 score units, yielding a small deterioration of 5.0%. Overall, this metric is only mildly affected by the change from asymmetric to symmetric weighting.
- **RG**: The time within the desired range increases for BIS, from 62.7 to 66.2 score units, corresponding to an improvement of 5.3%. For NOX, RG decreases slightly from 62.4 to 61.8 score units, which represents a minor reduction of 1.0%. Compared with the range-based configurations, the inversion-based tracking strategy increases the time in range for BIS and maintains a similar range performance for NOX. Regarding the time in range, the mean and median values are similar, suggesting approximately symmetric distributions. The dispersion remains acceptable, although it is slightly larger for NOX.
- **LRG**: For BIS, the symmetric tracking strategy reduces the percentage of samples below the lower range from 17.1 to 14.5 score units, which represents an improvement of 17.9%. For NOX, LRG increases slightly from 31.9 to 32.2 score units, corresponding to a small deterioration of 0.9%. Compared with the range-based configurations, the tracking strategy generally reduces the lower-range occupancy, especially under the symmetric weighting configuration.

**Table 6:** Monitor metrics in medians for Experiment 2, symmetric tracking (ESynPID) vs. asymmetric tracking.

Exp.2		MDPE		GS		NADIR		HRG		RG		LRG	
Weighting	Mode	BIS	NOX	BIS	NOX	BIS	NOX	BIS	NOX	BIS	NOX	BIS	NOX
Asymmetric	Range	−12.5	16.0	29.0	75.1	21.4	6.9	20.1	5.9	63.7	59.7	16.2	34.4
Symmetric	Range	−13.0	16.5	28.9	76.2	16.8	6.8	20.7	6.0	62.9	59.2	16.5	34.8
Asymmetric	Tracking	−13.0	12.8	29.6	54.2	16.4	7.3	20.2	5.7	62.7	62.4	17.1	31.9
Symmetric	Tracking	−10.0	18.8	26.6	61.1	18.3	8.2	19.3	6.0	66.2	61.8	14.5	32.2
Sym. Trac. vs. Asym. Track.		−30.0%	32.0%	−11.3%	11.2%	10.8%	11.0%	−4.7%	5.0%	5.3%	−1.0%	−17.9%	0.9%

**Table 7:** Primary metrics in mean (SD) for Experiment 2, symmetric tracking (ESynPID) vs. asymmetric tracking.

Exp.2		GS		NADIR		RG	
Weighting	Mode	BIS	NOX	BIS	NOX	BIS	NOX
Asymmetric	Range	30.6 (6.4)	85.2 (59.6)	29.9 (3.1)	15.8 (4.8)	63.7 (4.0)	59.1 (13.0)
Symmetric	Range	31.3 (8.0)	87.4 (57.9)	29.3 (4.4)	15.7 (4.5)	62.9 (4.2)	59.2 (13.3)
Asymmetric	Tracking	31.1 (7.2)	77.8 (77.4)	28.9 (5.6)	15.2 (4.9)	62.7 (5.4)	62.4 (13.9)
Symmetric	Tracking	27.6 (7.3)	74.8 (60.2)	29.3 (5.6)	15.5 (4.5)	66.2 (4.8)	61.9 (12.6)

### 3.3. Experiment 3: Setpoint Effect Under Asymmetric Weighting: Regulator vs. Tracking

Table 8 presents the performance evaluation of the asymmetric weighting strategy under different setpoint-generation modes, based on the median values obtained across a cohort of 12 virtual patients. Table 9 reports the primary performance metrics (see Section 2.10) as mean values and standard deviations. In this experiment, the asymmetric regulator configuration is used as the reference case, whereas the asymmetric tracking configuration is evaluated to quantify the isolated effect of the tracking mechanism when the weighting strategy remains unchanged.

- **MDPE**: For BIS, both configurations remain below the target value of 50 MU. However, the tracking configuration reduces the deviation from −14 MU to −13 MU, placing the BIS response 1 MU closer to the target, which corresponds to the 8% improvement reported in the table. For NOX, the monitor remains above the target value of 30 MU, but the deviation increases from 9.5 MU to 12.75 MU. This indicates that the NOX response moves farther from the target, with a deterioration of approximately 25%.
- **GS**: The global score improves for both monitors when the tracking mode is used. For BIS, GS decreases from 31.2 to 29.6 score units, which represents an improvement of approximately 5%. For NOX, GS decreases from 57.9 to 54.2 score units, corresponding to an improvement of approximately 7%. This suggests that, under asymmetric weighting, the tracking mechanism improves the overall response quality.
  If ESynPID were implemented with asymmetric weighting, the mean BIS value would remain close to the median, with a standard deviation of 7.2. However, the mean NOX value is 23.6 MU higher than the median, indicating a distribution skewed toward higher values. In addition, the large NOX standard deviation (77.4 MU) suggests that at least one virtual patient is poorly controlled under this configuration.
- **NADIR**: The tracking configuration reduces the NADIR metric for both monitors. For BIS, the value decreases from 21.4 to 16.4 score units, corresponding to a reduction of approximately 31%. For NOX, the value decreases from 8.2 to 7.3 score units, corresponding to a reduction of approximately 12%. When the asymmetric weighting strategy is preserved, the mean BIS NADIR under Tracking is 12.5 MU higher than the median, whereas for NOX the difference is 7.9 MU. This indicates that the distributions are shifted toward higher monitor values, although the dispersion remains acceptable, with standard deviations of 5.6 MU for BIS and 4.9 MU for NOX.
- **HRG**: The percentage of samples above the upper range is reduced for both monitors. For BIS, HRG decreases from 22.6 to 20.2 score units, representing an improvement of approximately 12%. For NOX, HRG decreases from 6.1 to 5.7 score units, corresponding to an improvement of approximately 7%.
- **RG**: The time within the desired range slightly increases for both monitors. For BIS, RG increases from 61.0 to 62.7 score units, corresponding to an improvement of approximately 3%. For NOX, RG remains practically unchanged, increasing only from 62.2 to 62.4 score units. Regarding the time in range, the mean and median values are similar, approximately symmetric distributions. The dispersion is acceptable, although slightly larger for NOX. Therefore, the time-in-range metric is not strongly affected by changing only the setpoint-generation mode under asymmetric weighting.
- **LRG**: The percentage of samples below the lower range increases slightly with the tracking configuration. For BIS, LRG increases from 16.4 to 17.1 score units, corresponding to a deterioration of approximately 4%. For NOX, the increase is minimal, from 31.7 to 31.9 score units, corresponding to approximately 1%.

**Table 8:** Monitor metrics in medians for Experiment 3, regulator [2] vs. tracking under asymmetric weighting.

Exp.3		MDPE		GS		NADIR		HRG		RG		LRG	
Weighting	Mode	BIS	NOX	BIS	NOX	BIS	NOX	BIS	NOX	BIS	NOX	BIS	NOX
Asymmetric	Range	−12.5	16.0	29.0	75.1	21.4	6.9	20.1	5.9	63.7	59.7	16.2	34.4
Asymmetric	Regulator	−14.0	9.5	31.2	57.9	21.4	8.2	22.6	6.1	61.0	62.2	16.4	31.7
Asymmetric	Tracking	−13.0	12.8	29.6	54.2	16.4	7.3	20.2	5.7	62.7	62.4	17.1	31.9
Tracking vs. Regulator		−8.0%	25.0%	−5.0%	−7.0%	−31.0%	−12.0%	−12.0%	−7.0%	3.0%	0.0%	4.0%	1.0%

**Table 9:** Primary metrics in mean (SD) for Experiment 3, regulator [2] vs. tracking under asymmetric weighting.

Exp.3		GS		NADIR		RG	
Weighting	Mode	BIS	NOX	BIS	NOX	BIS	NOX
Asymmetric	Range	30.6 (6.4)	85.2 (59.6)	29.9 (3.1)	15.8 (4.8)	63.7 (4.0)	59.1 (13.0)
Asymmetric	Regulator	32.8 (5.7)	77.2 (73.9)	29.1 (4.0)	15.2 (4.2)	60.9 (4.8)	62.2 (12.7)
Asymmetric	Tracking	31.1 (7.2)	77.8 (77.4)	28.9 (5.6)	15.2 (4.9)	62.7 (5.4)	62.4 (13.9)

### 3.4. Experiment Summary

This section summarises the monitor profiles, target trajectories, and drug perfusion patterns obtained when comparing ESynPID with the original SynPID strategy.

**Figure 4:**
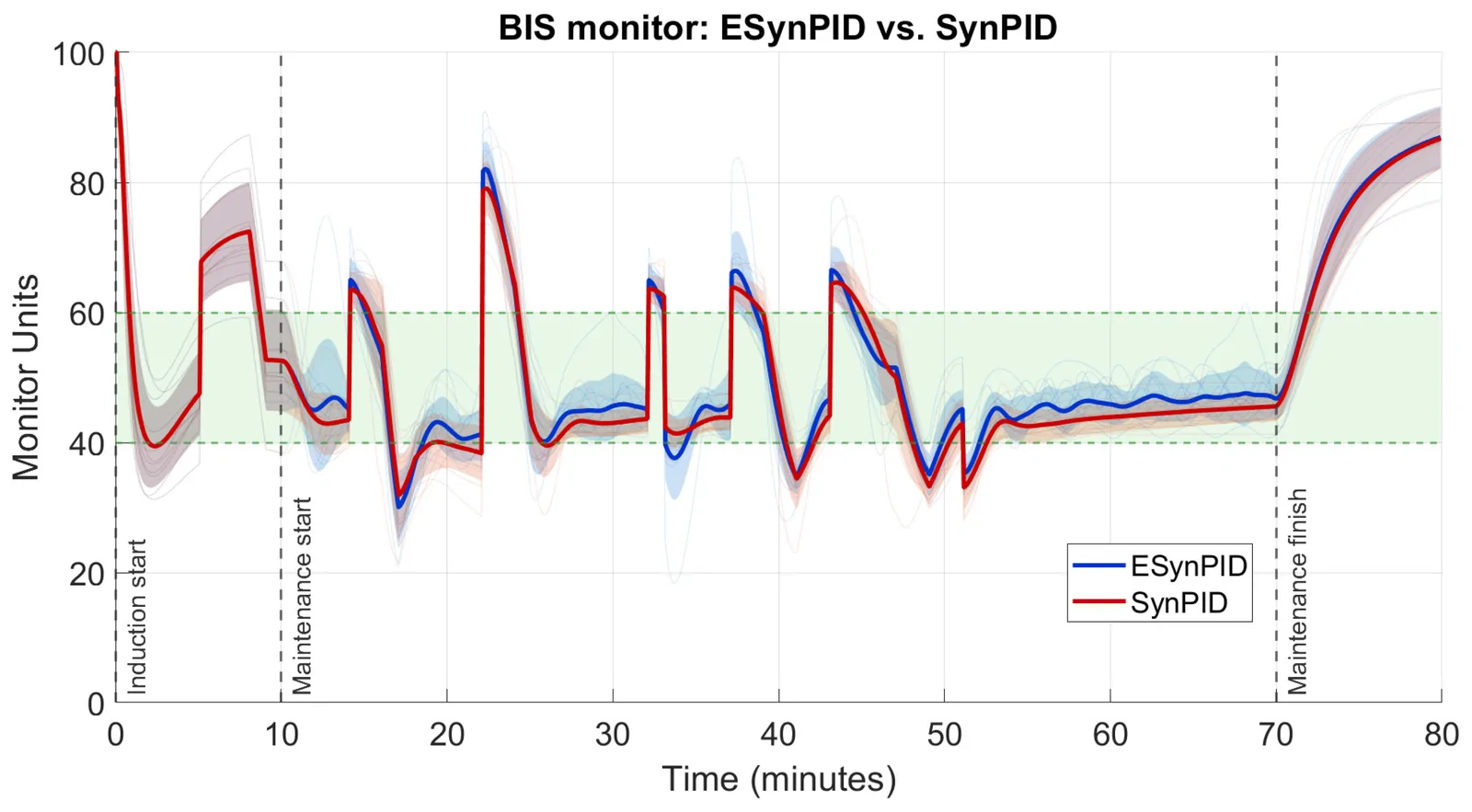
ESynPID vs. SynPID BIS response. The green band indicates a safe range.

**Figure 5:**
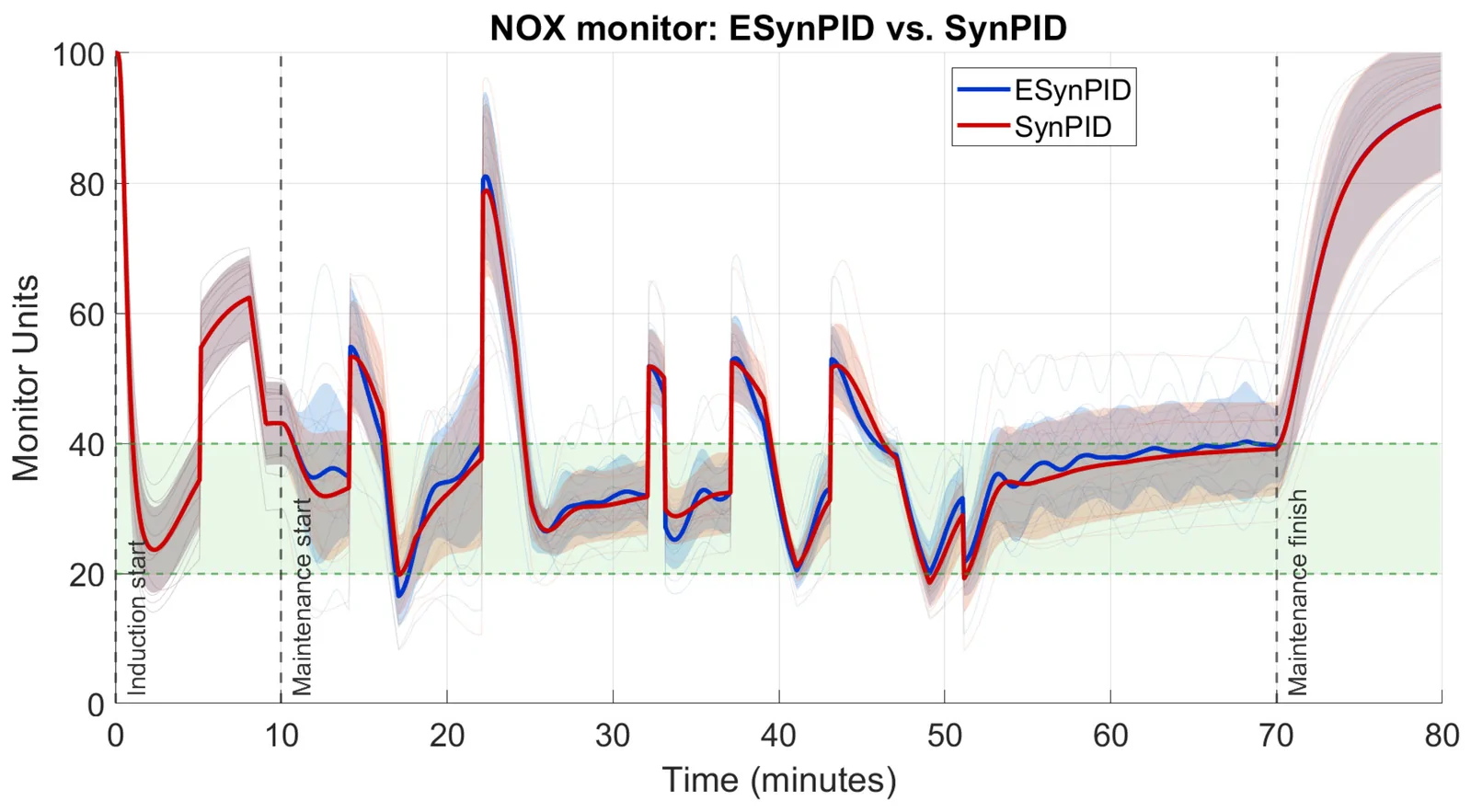
ESynPID vs. SynPID NOX response. The green band indicates a safe range.

Figure 4 and Figure 5 show the BIS and NOX monitor responses from the beginning of induction to the end of recovery. During the induction phase, both strategies exhibit the same behaviour because the OLP protocol is applied in both cases. Differences appear after the beginning of the maintenance phase, where ESynPID generally produces higher monitor values during quasi-steady periods and around the application or withdrawal of surgical stimuli.

**Figure 6:**
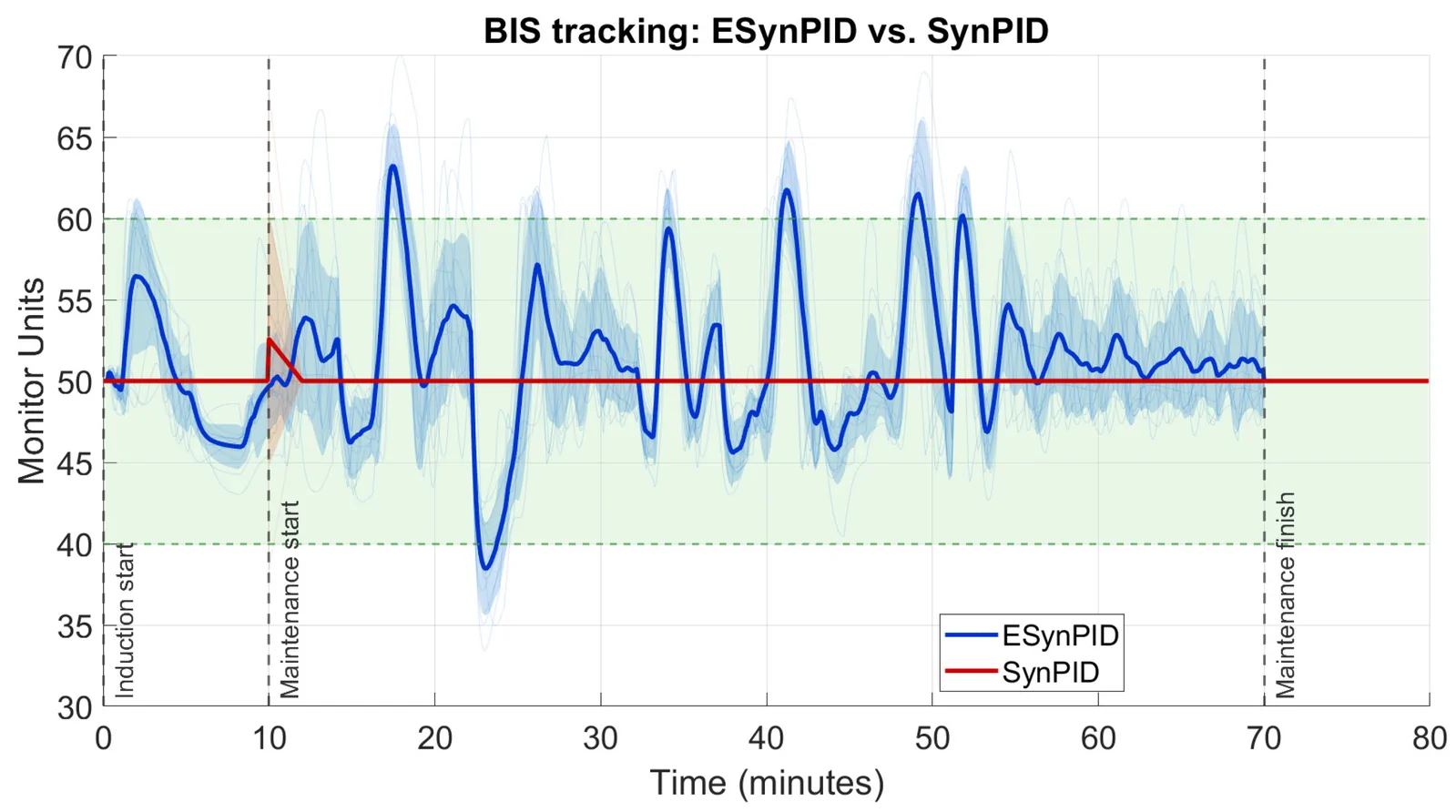
ESynPID vs. SynPID BIS setpoint profile. The green band indicates a safe range.

**Figure 7:**
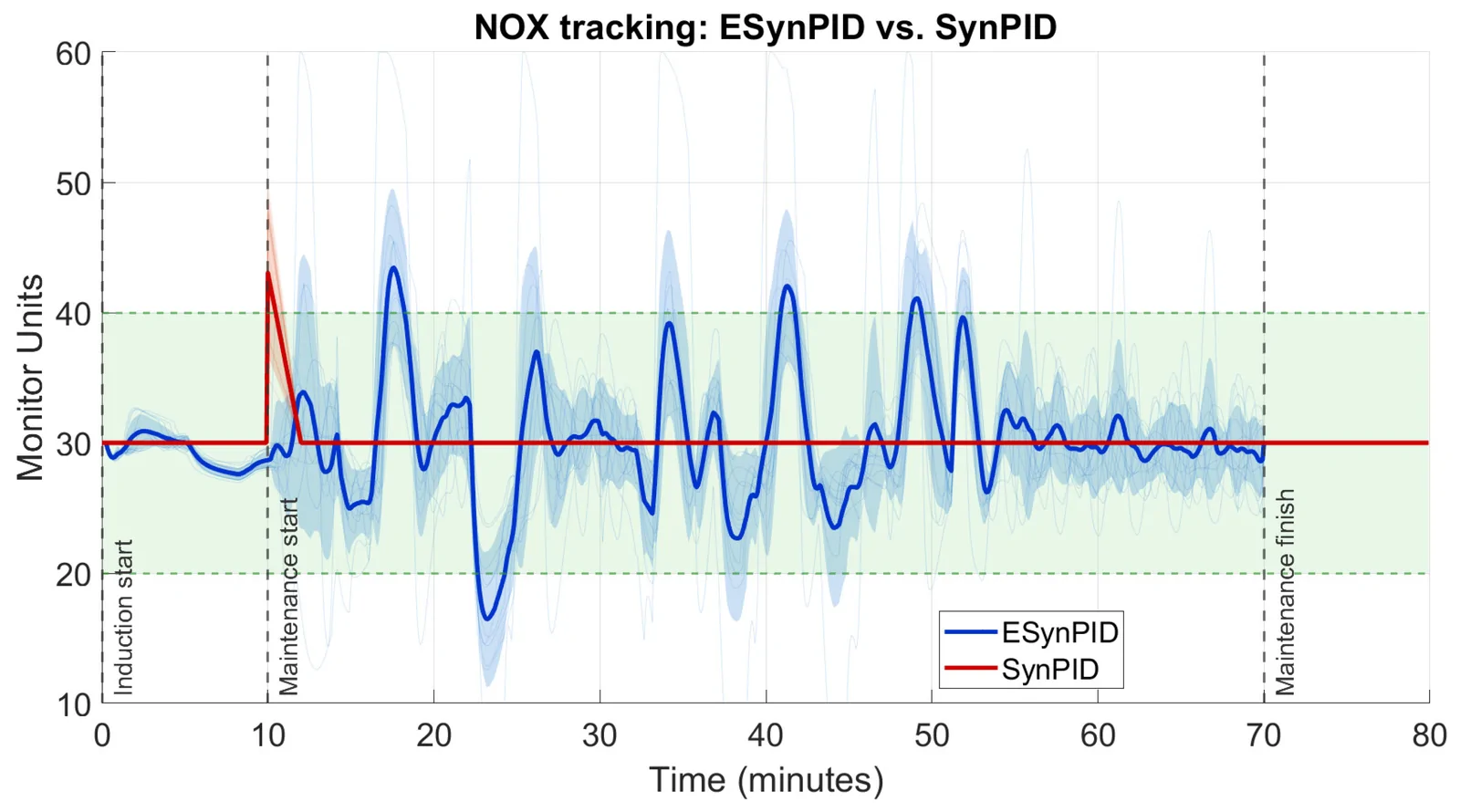
ESynPID vs. SynPID NOX setpoint profile. The green band indicates a safe range.

Figure 6 and Figure 7 compare the BIS and NOX setpoint profiles. During induction, the ESynPID setpoint varies as a result of the retroactive PID state-alignment procedure; however, this does not affect the patient because the system is still operating under OLP. After the OLP–CLP switching instant, the difference between both approaches becomes clear: SynPID keeps a mostly constant setpoint, whereas ESynPID generates a variable tracking signal that evolves according to the monitor response.

**Figure 8:**
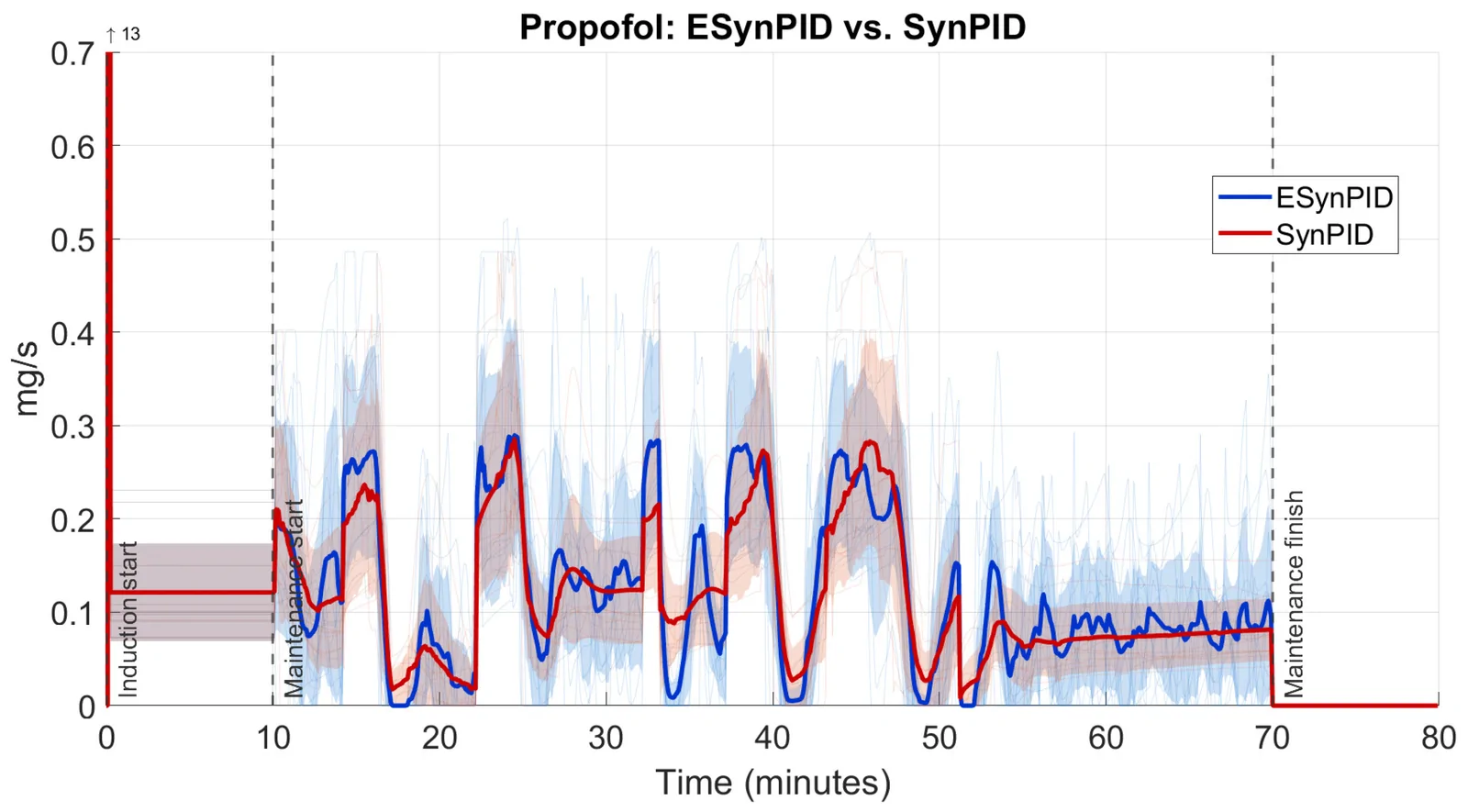
ESynPID vs. SynPID propofol perfusion profile.

**Figure 9:**
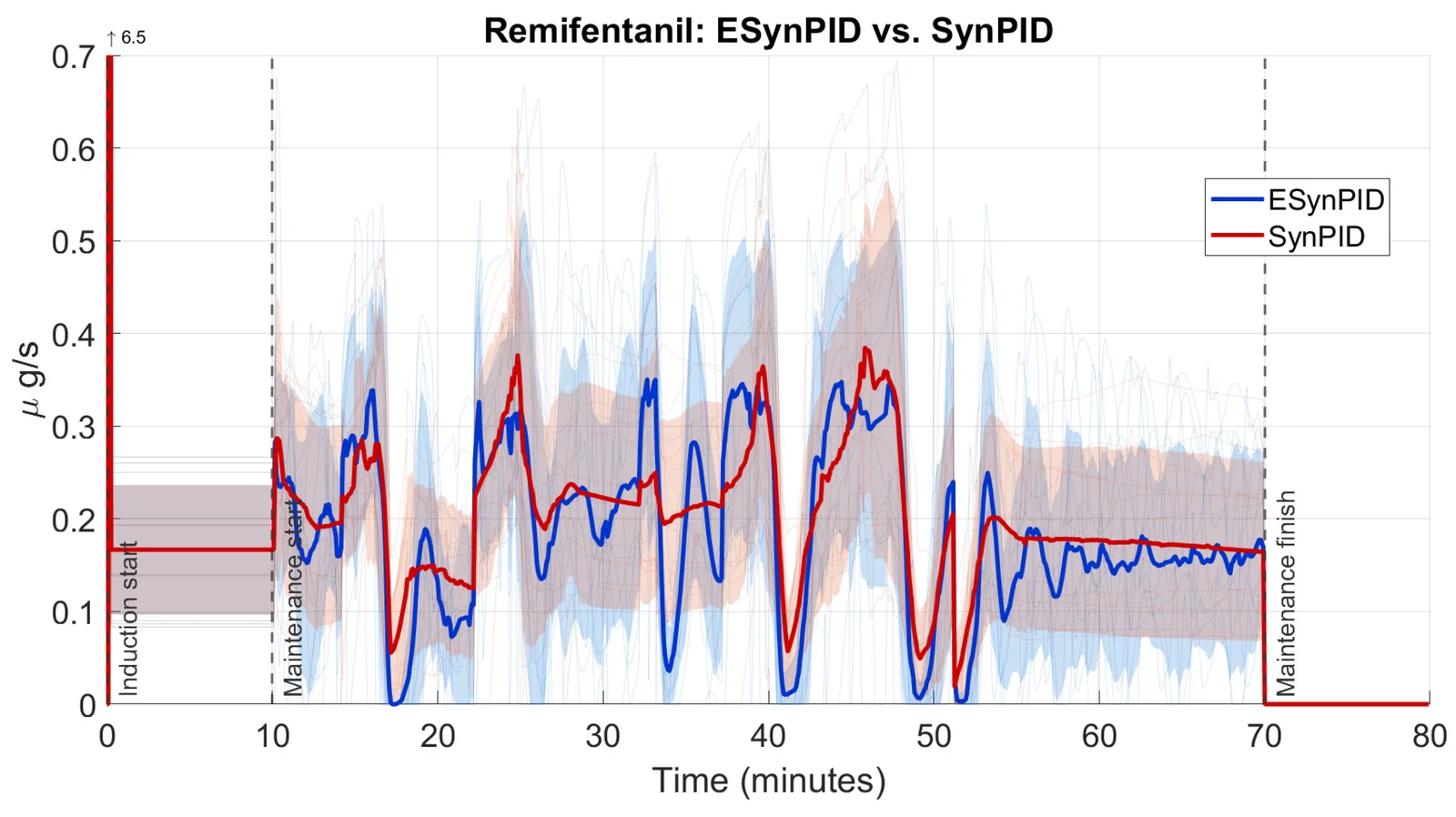
ESynPID vs. SynPID remifentanil perfusion profile.

Figure 8 and Figure 9 show the propofol and remifentanil perfusion profiles. During induction, both perfusions remain constant and practically identical because only OLP is active. Once the maintenance phase starts, clear differences appear in both the amplitude and timing of the perfusion changes. ESynPID introduces more pronounced variations around the surgical stimuli, reflecting the combined effect of the symmetric weighting strategy and the tracking mechanism.

Figure 8 shows the initial propofol induction bolus of 13 mg/s. This value is identical for both SynPID and ESynPID, as the induction phase is governed by the Open-Loop Protocol (OLP).

Figure 9 shows the initial remifentanil induction bolus of 6.5 µg/s. This value is identical for both SynPID and ESynPID, as the induction phase is controlled by the Open-Loop Protocol (OLP).

Table 10 compares the drug administration and Total Variation (TV) obtained with ESynPID and SynPID for the 12 virtual patients. Regarding the total amount of drug administered during the maintenance phase, ESynPID shows a mixed response. The mean RMF consumption is reduced from 624.3 to 582.9 µg, corresponding to a 7.11% decrease compared with SynPID. In contrast, the mean PPF administration increases from 374.9 to 390.5 mg, representing a 4.00% increase. A less favourable behaviour of ESynPID is observed in terms of Total Variation (TV). The mean TV increases from 3.61 to 7.41 mg/s^2^ for PPF and from 4.12 to 9.15 µg/s^2^ for RMF, corresponding to relative increases of 51.26% and 54.98%, respectively. Moreover, the standard deviation of TV is also higher with ESynPID, particularly for RMF (3.7 versus 1.9 µg/s^2^), indicating greater inter-patient variability in the control effort. Therefore, ESynPID does not outperform SynPID in terms of infusion smoothness; instead, the varying-target strategy results in more pronounced adjustments to the infusion rates.

**Table 10:** Amount of drug administered and Total Variation (TV) for ESynPID vs. SynPID.

VP No.	ESynPID				SynPID			
	Amount		Total Variation		Amount		Total Variation	
	PPF (mg)	RMF (µg)	PPF (mg/s^2^)	RMF (µg/s^2^)	PPF (mg)	RMF (µg)	PPF (mg/s^2^)	RMF (µg/s^2^)
1	457	402	7.60	4.55	441	415	4.23	2.29
2	302	326	5.11	4.62	306	332	2.47	2.20
3	318	313	7.27	6.13	300	345	3.40	2.76
4	642	606	10.45	4.78	634	631	4.77	2.15
5	225	783	3.53	9.05	212	847	1.55	3.68
6	365	358	9.10	6.89	324	417	4.07	2.74
7	276	913	5.67	15.30	239	1022	2.35	5.72
8	309	972	4.32	10.79	296	1020	1.75	4.18
9	528	445	14.14	12.87	452	554	7.73	5.70
10	552	163	10.98	11.70	588	130	6.53	8.23
11	402	941	5.54	13.08	408	954	2.05	5.27
12	310	773	5.25	10.01	297	825	2.46	4.49
Mean	**390.5**	**582.9**	**7.41**	**9.15**	**374.9**	**624.3**	**3.61**	**4.12**
Relative (%)	4.00%	−7.11%	51.26%	54.98%				
SD	128.1	283.2	3.2	3.7	132.9	303.4	1.9	1.9
Relative (%)	−3.76%	−7.15%	38.70%	49.83%				

## 4. Discussion

### 4.1. Implementation and Design Considerations

The proposed controller was formulated in discrete time to match the sampled implementation of the virtual patient, whose PK–PD models are numerically propagated using ode15s. This formulation also allows the controller, target generator, weighting functions, safety module, quantisation stage, and pump model to be consistently represented within the same sampled-data framework.

Several design choices in ESynPID could be further explored. First, the OLP modulation could be extended by using the tracking signal generated by the inversion mechanism. In the current implementation, the OLP modulator mainly switches between 0 and 1 according to the monitor value and the safe-range threshold. A normalised tracking-dependent modulation could provide a smoother transition between open-loop and closed-loop contributions.

Regarding the PID discretisation, the integral action was approximated using the trapezoidal rule, whereas the derivative action was implemented through a discrete difference with a first-order low-pass filter. Other discretisation schemes, such as rectangular integration or higher-order digital derivative filters, could also be evaluated. However, the adopted structure offers a reasonable compromise between numerical simplicity, noise attenuation, and reduced delay.

The inversion mechanism was also modified with respect to its nominal formulation. Since the direct inversion may cause the generated reference to lose its driving role and converge to the measured monitor value, the current monitor feedback term was replaced by the constant target value. In addition, the inversion gain was introduced to reduce the sensitivity of the target generator to changes in drug perfusion and to improve relative stability. Although this gain was tuned heuristically, alternative tuning criteria could be considered to reduce the dependence of the inversion on the discretisation and weighting terms.

The retroactive PID state-alignment procedure was necessary to reduce abrupt transients during the OLP–CLP transition. At the end of induction, the PID controllers are tuned and the controller is virtually replayed using the previous monitor trajectories. The drug infusions generated during this retroactive stage are not applied to the patient; they are only used to initialise the internal controller states consistently with the monitored dynamics. This procedure prepares the controller before activating CLP with tracking at the beginning of maintenance. A shorter replay window could be explored in future work, provided that it is long enough for the internal states to reach a consistent condition before switching.

### 4.2. Effect of Tracking and Weighting Strategies on Controller Performance

The three experiments provide complementary evidence regarding the effects of the proposed ESynPID modifications. In Experiment 1, the complete ESynPID configuration improves the BIS response with respect to the original SynPID regulator. However, this improvement is not equally reflected in the NOX response. The analysis of the mean and standard deviation values shows that the NOX distribution can be skewed toward higher values, suggesting that one or more virtual patients may remain poorly controlled. Therefore, the tracking-oriented target generation improves some aspects of the closed-loop response, but it does not guarantee a uniform improvement across all patients or all monitors.

Experiment 2 isolates the effect of changing the weighting strategy from asymmetric to symmetric while preserving the tracking configuration. The results indicate that symmetric weighting contributes to improving the BIS response and slightly modifies the NADIR behaviour. Nevertheless, the NOX response remains more sensitive to patient-dependent variability. In particular, the difference between median and mean NOX values, together with the larger dispersion, suggests that the weighting modification alone is not sufficient to compensate for poorly controlled cases. This indicates that the symmetric weighting strategy strengthens the controller action, but its effect depends on the monitor and on the underlying patient response.

Experiment 3 evaluates the isolated effect of the tracking mechanism under asymmetric weighting. The results show that changing only the setpoint generation mode provides limited benefits. Although some metrics improve under tracking, the mean–median differences and the dispersion values indicate that the effect is not strong enough to consistently enhance the response of both monitors. This behaviour motivated the complete ESynPID formulation, in which tracking is combined with the symmetric weighting strategy and the updated control-to-range module.

Overall, the results suggest that the proposed tracking mechanism mainly modifies the dynamic behaviour of the controller, whereas the weighting strategy determines how assertively the controller reacts to deviations from the target. However, the time-in-range metric remains only mildly affected in the three experiments. This indicates that further improvements in range maintenance may require modifications beyond the target generator, such as changes in the controller tuning, the anti-windup window, the pump-emulation limits, or the auxiliary support modules. These observations also highlight that ESynPID improves adaptability, but its performance remains influenced by patient variability and by the tuning of the underlying PID controllers. Across the evaluated configurations, including symmetric and asymmetric weighting strategies, no substantial differences were observed in the RG metric for either BIS or NOX. This suggests that the proposed modifications have a limited effect on the percentage of time that the monitors remain within the desired range. Regarding NADIR, the differences were less relevant for NOX, indicating that this metric is only weakly affected by the tested control variants. In Experiment 3, the changes observed in the primary metrics were minor; therefore, they do not provide strong evidence of a relevant performance improvement when only the setpoint-generation mode is modified under asymmetric weighting.

### 4.3. Perfusion Profiles and Control–Effort Trade-Off

The perfusion profiles in Figure 8 and Figure 9 show that ESynPID anticipates drug delivery when surgical stimuli appear and reduces perfusion more sharply when they disappear. However, this anticipated action does not affect the monitors as strongly as expected, mainly because of the slow pharmacokinetic dynamics of the virtual patient models.

The drug-administration results reported in Table 10 show that ESynPID produces a mixed response during the maintenance phase. The mean RMF consumption is reduced compared with SynPID, whereas the mean PPF administration increases. This indicates that the varying-target strategy does not uniformly reduce the total amount of administered drugs, but rather redistributes the control effort between the two drug channels.

A less favourable behaviour is observed in terms of Total Variation (TV). The mean TV increases for both PPF and RMF under ESynPID, and the standard deviation of TV is also higher, particularly for RMF. This indicates greater inter-patient variability in the control effort and confirms that ESynPID does not outperform SynPID in terms of infusion smoothness. Instead, the varying-target strategy results in more pronounced adjustments to the infusion rates.

These results highlight an important trade-off associated with the proposed strategy. The enhanced tracking capability of ESynPID does not necessarily translate into either lower drug administration or smoother control action for both drugs. In particular, the substantial increase in TV indicates that the improved tracking behaviour reported for ESynPID is achieved at the expense of greater control activity. Therefore, the benefits of ESynPID should be assessed jointly with the clinical performance metrics, rather than from the perfusion metrics alone.

### 4.4. Scope of the Rocuronium Channel Analysis

The closed-loop performance metrics for the rocuronium channel (C33) were not included in the comparative analysis because the channel experiences no external disturbances during the evaluated scenario. Under these steady-state conditions, the error-based time-varying reference design is not stimulated, as the control algorithm naturally minimises the tracking error without requiring dynamic adjustments in the reference signal. As a result, the control response for this channel remains identical across the evaluated schemes.

### 4.5. Scope of the Comparative Analysis

For the ESynPID versus SynPID performance comparison, only monitor-based metrics were considered, as infusion-related metrics inherently yield higher values due to the use of the tracking mechanism. It is worth emphasising that the comparative analysis in this study focuses strictly on evaluating the incremental benefits of the proposed error-driven time-varying reference scheme against its baseline SynPID structure. A broader benchmark against other state-of-the-art control strategies was previously established in [2]. Furthermore, controllers designed purely for regulatory tasks, i.e., disturbance rejection at a fixed operating point, are functionally distinct from tracking-oriented controllers that dynamically modify the reference signal. Consequently, a direct quantitative comparison between regulation and tracking frameworks was omitted to avoid methodologically inconsistent evaluations.

### 4.6. Robustness Considerations

It is important to clarify that the proposed ESynPID strategy is not an online model-based controller. The PK–PD model is used to construct the virtual patients, propagate the simulated physiological response, and support the offline tuning procedure. However, during closed-loop operation, ESynPID does not require access to the internal PK–PD states or to the exact patient model parameters. Instead, the controller uses the sampled monitor outputs, the generated targets, the weighting functions, the Range–Control correction, and the drug perfusion feedback.

Therefore, robustness should be interpreted in the context of the tested virtual-patient cohort, the selected surgical stimuli, and the bounded implementation constraints. The results indicate that the proposed strategy can modify the perfusion profile and improve the response of some monitored variables under these conditions. Nevertheless, the controller performance may still depend on the quality of the offline tuning and on the representativeness of the virtual patients used during the design stage. Large model mismatch, unmodelled patient dynamics, or clinical conditions not represented in the virtual cohort may require retuning, adaptation, or additional supervisory mechanisms.

This limitation is consistent with the nature of PID-based anaesthesia control: robustness is partly provided by feedback and bounded control actions, but the performance level remains influenced by patient variability and by the selected tuning procedure.

## 5. Conclusions

This work presented an enhanced synergistic PID strategy, denoted as ESynPID, for multivariable drug delivery in closed-loop general anaesthesia. The proposed architecture extends the previous SynPID formulation by incorporating a PID-inversion-based target generator, a symmetric setpoint feedforward weighting strategy, and an updated symmetric control-to-range module. These elements were integrated within a sampled-data framework that also includes pump emulation, phase switching, and pharmacokinetic– pharmacodynamic virtual patient models.

The proposed formulation allows the controller to move from a regulation-oriented scheme based on fixed targets toward a tracking-oriented strategy where the setpoint evolves according to the monitored response and the perfusion feedback. This provides a more active control structure during the maintenance phase and allows the controller to react more assertively to the appearance and disappearance of surgical stimuli.

The experimental results show that ESynPID modifies the drug perfusion profiles with respect to the original SynPID strategy. In particular, the tracking mechanism and the symmetric weighting strategy produce more dynamic perfusion patterns, especially around stimulus transitions. This behaviour improves the response of the hypnotic monitor, while also revealing a trade-off in the nociception monitor. Therefore, the proposed symmetry and tracking mechanisms should be interpreted as a way to increase adaptability and robustness, although their effect depends on the monitored variable and on the interaction between drug dynamics and controller action.

The comparison between asymmetric and symmetric configurations also indicates that symmetry is not uniformly beneficial for all monitors. While it may improve the response associated with hypnosis, the asymmetric formulation can provide a more balanced behaviour when both hypnosis and nociception are considered simultaneously. This suggests that future developments should explore adaptive or monitor-dependent weighting laws instead of using a fixed symmetric structure for all control channels.

Finally, the proposed ESynPID architecture provides a consistent framework for combining variable target generation, multivariable PID control, range correction, and pump emulation in closed-loop anaesthesia simulation. Future work should focus on refining the inversion-gain tuning procedure, improving the interaction between the target generator and the Range–Control module, and evaluating the proposed approach under broader patient variability, uncertainty, and clinically realistic disturbance scenarios.

## Data Availability

The data are not publicly available because they correspond to MATLAB 2024b simulation files, model parameters, and processed results generated for the present study.

## Abbreviations

The following abbreviations are used in this manuscript:

AI	Artificial Intelligence
BIS	Bispectral Index
CLP	Closed-Loop Protocol
CTRM	Control-to-Range Module
ESynPID	Enhanced Synergistic PID
FOPI	Fractional-Order Proportional–Integral controller
FOPID	Fractional-Order Proportional–Integral–Derivative controller
GS	Global Score
HRG	Higher Range
IAE	Integral Absolute Error
ISA-PID	International Society of Automation PID
LRG	Lower Range
MDPE	Median Performance Error
MIMO	Multiple-Input Multiple-Output
MISO	Multiple-Input Single-Output
MU	Monitorisation Unit
NMB	Neuromuscular Blockade
NOX	Nociception Index
OLP	Open-Loop Protocol
PD	Pharmacodynamics
PID	Proportional–Integral–Derivative controller
PK	Pharmacokinetics
PK–PD	Pharmacokinetic–Pharmacodynamic
PPF	Propofol
RCN	Rocuronium
RG	Range
RMF	Remifentanil
S/H	Sample-and-Hold
SynPID	Synergistic PID
TIVA	Total Intravenous Anaesthesia
VP	Virtual Patient
ZOH	Zero-Order Hold

## Appendix A. Stability Analysis of the ESynPID Topology

This appendix presents a Lyapunov-based stability analysis of the discrete-time ESynPID topology shown in Figure 1. The architecture includes the PID Inversor, the Error Generator, the controller, the Open Loop Modulator, the control-to-range module, the Phase Switching mechanism, the pump emulation block, and the sampled Virtual Patient. The S/H blocks provide the sampled open-loop perfusion $u_{ol}\left(k\right)$ and the sampled monitor vector y(k) from their continuous-time counterparts $u_{ol}\left(t\right)$ and y(t), respectively.

The monitor vector and the generated reference vector are defined as

(A1) $$y\left(k\right)={\left[\begin{array}{ccc} y_{1}\left(k\right) & y_{2}\left(k\right) & y_{3}\left(k\right) \end{array}\right]}^{⊤},\;r\left(k\right)={\left[\begin{array}{ccc} r_{1}\left(k\right) & r_{2}\left(k\right) & r_{3}\left(k\right) \end{array}\right]}^{⊤}.$$

The *Error Generator* produces the monitor error vectors (3).

The aggregate error vector is defined as

(A2) $$e\left(k\right)={\left[\begin{array}{ccc} e_{1}^{⊤}\left(k\right) & e_{2}^{⊤}\left(k\right) & e_{3}^{⊤}\left(k\right) \end{array}\right]}^{⊤}.$$

For each physiological monitor j∈{1,2,3}, the discrete-time weighted errors are given by (4). The filtered derivative error is computed through the first-order recursion:

(A3) $$e_{F,j}\left(k\right)=p_{F,ij}e_{F,j}\left(k-1\right)+K_{F,ij}e_{D,j}\left(k\right),$$

where $0<p_{F,ij}<1$ and $K_{F,ij}>0$. Therefore, the filtered derivative subsystem is exponentially stable.

The weighting module guarantees that the proportional and derivative weights remain bounded:

(A4) $$1\leq \beta_{j}\left(k\right)\leq B_{j},\;1\leq \gamma_{j}\left(k\right)\leq G_{j},\;j\in \left\{1,2,3\right\}.$$

Let $\eta \left(k\right)\in R^{5}$ collect the five integral states of the ISA-PID controllers:

(A5) $$\eta \left(k\right)={\left[\begin{array}{ccccc} I_{11}\left(k\right) & I_{12}\left(k\right) & I_{21}\left(k\right) & I_{22}\left(k\right) & I_{33}\left(k\right) \end{array}\right]}^{⊤}.$$

The integral action includes an anti-windup saturation, so that

(A6) $$|I_{ij}\left(k\right)|\leq {\bar{I}}_{ij},\;\left(i,j\right)\in S.$$

The augmented controller state is then defined as

(A7) $$\chi \left(k\right)={\left[\begin{array}{cc} e^{⊤}\left(k\right) & \eta^{⊤}\left(k\right) \end{array}\right]}^{⊤}.$$

To explicitly account for the dynamics introduced by the PID Inversor, the inverter state is defined as

(A8) $$\xi_{r}\left(k\right)={\left[\begin{array}{cccccc} r_{1}\left(k\right) & r_{1}\left(k-1\right) & r_{2}\left(k\right) & r_{2}\left(k-1\right) & r_{3}\left(k\right) & r_{3}\left(k-1\right) \end{array}\right]}^{⊤}.$$

Then, the extended controller–inverter state is written as

(A9) $$\bar{\chi }\left(k\right)={\left[\begin{array}{cc} \chi^{⊤}\left(k\right) & \xi_{r}^{⊤}\left(k\right) \end{array}\right]}^{⊤}.$$

According to Figure 1, the *controller* generates the closed-loop control contribution $u_{c}\left(k\right)$, while the *control-to-range module* generates the correction term $u_{cr}\left(k\right)$. These signals are combined and, together with the contribution of the *Open Loop Modulator*, are routed through the *Phase Switching* mechanism. The resulting sampled drug infusion vector u(k) is processed by the *pump emulation* block, which includes the safety mechanism, quantifier, and ZOH stage, yielding the continuous-time infusion u(t) applied to the virtual patient.

The safety mechanisms, which are included into the pump emulation, impose bounded and nonnegative infusion signals:

(A10) $$0\leq u_{i}\left(k\right)\leq u_{Hi},\;i\in \left\{1,2,3\right\},$$

where $u_{Hi}$ is the maximum admissible infusion rate of the *i*-th drug delivery channel.

The interaction between the PID Inversor and the control-to-range module can be described through the feedback perfusion signal used by the inverter. For each drug channel, this signal is given by

(A11) $$u_{bi}\left(k\right)=\sum_{j\in J_{i}}\left(u_{c,ij}\left(k\right)+u_{cr,ij}\left(k\right)\right),$$

where $J_{1}=\left\{1,2\right\}$ for PPF, $J_{2}=\left\{1,2\right\}$ for RMF, and $J_{3}=\left\{3\right\}$ for RCN. Therefore, the PID Inversor does not interact with the control-to-range module through an independent loop, but through the bounded feedback perfusion $u_{b}\left(k\right)$.

The final inversion law can be written as the second-order recursion:

(A12) $$r_{i}\left(k\right)=a_{1i}r_{i}\left(k-1\right)+a_{2i}r_{i}\left(k-2\right)+b_{i}y_{Ti}-g_{i}\Delta u_{bi}\left(k\right),$$

with

(A13) $$a_{1i}=-\frac{K_{4i}}{K_{3i}},\;a_{2i}=-\frac{K_{5i}}{K_{3i}},\;b_{i}=\frac{K_{0i}+K_{1i}+K_{2i}}{K_{3i}},\;g_{i}=\frac{K_{ui}}{K_{3i}}.$$

For the inverter dynamics, it is defined that

(A14) $$\xi_{r,i}\left(k\right)=\left[\begin{array}{c} r_{i}\left(k\right)\\ r_{i}\left(k-1\right) \end{array}\right].$$

Then,

(A15) $$\xi_{r,i}\left(k+1\right)=A_{r,i}\xi_{r,i}\left(k\right)+B_{r,i}\left(b_{i}y_{Ti}-g_{i}\Delta u_{bi}\left(k+1\right)\right),$$

where

(A16) $$A_{r,i}=\left[\begin{array}{cc} a_{1i} & a_{2i}\\ 1 & 0 \end{array}\right],\;B_{r,i}=\left[\begin{array}{c} 1\\ 0 \end{array}\right].$$

Thus, a sufficient condition for boundedness of the PID Inversor is

(A17) $$\rho \left(A_{r,i}\right)<1,\;i\in \left\{1,2,3\right\},$$

where ρ(·) denotes the spectral radius. Under (A17), the PID Inversor is BIBO stable with respect to the bounded input $\Delta u_{bi}\left(k\right)$.

On the other hand, the control-to-range module is a piecewise affine and saturated correction law. Since the monitor outputs are bounded within their physiological ranges and the weighting functions are bounded by (A4), there exists a finite constant ${\bar{u}}_{cr,i}>0$ such that

(A18) $$|u_{cr,i}\left(k\right)|\leq {\bar{u}}_{cr,i},\;i\in \left\{1,2,3\right\}.$$

Consequently, if the ISA-PID internal signals are bounded, then the feedback perfusion used by the inverter is also bounded:

(A19) $$|u_{bi}\left(k\right)|\leq {\bar{u}}_{b,i},\;\left|\Delta u_{bi}\left(k\right)\right|\leq 2{\bar{u}}_{b,i}.$$

Equations (A12)–(A19) show that the PID Inversor–control-to-range module interconnection is a bounded-gain feedback interconnection. In practice, the inversion gain $K_{ui}$ is tuned to reduce the gain $g_{i}$ and avoid excessive amplification of $\Delta u_{bi}\left(k\right)$. Therefore, the interaction between both blocks remains bounded provided that the inverter recursion is Schur stable and the inversion gain is selected so that the induced feedback gain remains below the closed-loop admissible bound.

Around a desired operating point, the discrete-time ESynPID interconnection can be represented as

(A20) $$\bar{\chi }\left(k+1\right)=F_{c}\left(\bar{\chi }\left(k\right),{\tilde{x}}_{p}\left(k\right),u_{ol}\left(k\right),u_{b}\left(k\right),u_{cr}\left(k\right)\right),$$

(A21) $${\tilde{x}}_{p}\left(k+1\right)=F_{p}\left({\tilde{x}}_{p}\left(k\right),u\left(k\right),d\left(k\right)\right),$$

where ${\tilde{x}}_{p}\left(k\right)$ denotes the deviation of the sampled Virtual Patient state from the equilibrium, $u_{ol}\left(k\right)$ is the sampled Open-Loop Protocol input, $u_{b}\left(k\right)$ is the feedback perfusion used by the PID Inversor, $u_{cr}\left(k\right)$ is the correction provided by the control-to-range module, u(k) is the sampled infusion selected by the Phase Switching mechanism, and d(k) represents bounded surgical stimuli.

A composite Lyapunov candidate is proposed as

(A22) $$V\left(k\right)=V_{e}\left(k\right)+V_{I}\left(k\right)+V_{r}\left(k\right)+V_{p}\left(k\right),$$

where

(A23) $$V_{e}\left(k\right)=\sum_{j=1}^{3}e_{j}^{⊤}\left(k\right)P_{j}e_{j}\left(k\right),\;P_{j}=P_{j}^{⊤}>0,$$

(A24) $$V_{I}\left(k\right)=\eta^{⊤}\left(k\right)\Lambda \eta \left(k\right),\;\Lambda =\Lambda^{⊤}>0,$$

(A25) $$V_{r}\left(k\right)=\xi_{r}^{⊤}\left(k\right)P_{r}\xi_{r}\left(k\right),\;P_{r}=P_{r}^{⊤}>0,$$

and

(A26) $$V_{p}\left(k\right)={\tilde{x}}_{p}^{⊤}\left(k\right)P_{p}{\tilde{x}}_{p}\left(k\right),\;P_{p}=P_{p}^{⊤}>0.$$

Since $0<p_{F,ij}<1$ in (5), the filtered derivative dynamics are stable. Moreover, the weighting functions are bounded by (A4), the integral states are bounded by (10), the drug infusions are bounded by (12), the control-to-range module is bounded by (A18), and the PID Inversor is BIBO stable under (A17). Therefore, all controller-side internal signals remain bounded for bounded monitor and reference signals.

Assuming that the sampled virtual patient is locally input-to-state stable around the operating point, there exist positive constants $\lambda_{c}$, $\lambda_{r}$, $\lambda_{p}$, $\rho_{ol}$, $\rho_{b}$, $\rho_{cr}$, and $\rho_{d}$ such that

(A27) $$\begin{array}{cc} \Delta V(k)=V(k+1)-V(k)\leq & -\lambda_{c}{∥\chi \left(k\right)∥}^{2}-\lambda_{r}∥\xi_{r}{\left(k\right)∥}^{2}-\lambda_{p}∥{\tilde{x}}_{p}{\left(k\right)∥}^{2}+\rho_{ol}{∥u_{ol}\left(k\right)∥}^{2}\\ \; & +\rho_{b}∥u_{b}{\left(k\right)∥}^{2}+\rho_{cr}∥u_{cr}{\left(k\right)∥}^{2}+\rho_{d}{∥d\left(k\right)∥}^{2}. \end{array}$$

Equation (A27) implies that the complete discrete-time ESynPID topology is input-to-state stable with respect to the bounded open-loop contribution, bounded inversion feedback perfusion, bounded Range–Control correction, bounded surgical disturbances, and bounded implementation effects introduced by pump emulation.

In particular, the term $\rho_{b}∥u_{b}{\left(k\right)∥}^{2}+\rho_{cr}{∥u_{cr}\left(k\right)∥}^{2}$ explicitly accounts for the interaction between the PID Inversor and the control-to-range module. The former receives $u_{b}\left(k\right)$ as inversion feedback, whereas the latter modifies $u_{b}\left(k\right)$ through $u_{cr}\left(k\right)$. Since both signals are bounded and the inverter dynamics satisfy the Schur condition (A17), their interconnection does not introduce unbounded internal dynamics.

Then, all internal controller variables, the requested drug infusion u(k), the continuous-time infusion u(t) applied by the pump emulation block, and the monitor error vectors $e_{1}\left(k\right)$, $e_{2}\left(k\right)$, and $e_{3}\left(k\right)$ remain bounded.

In the nominal case, i.e., in the absence of surgical disturbances and implementation perturbations, (A27) reduces to

(A28) $$\Delta V\left(k\right)\leq -\lambda_{c}{∥\chi \left(k\right)∥}^{2}-\lambda_{r}{∥\xi_{r}\left(k\right)∥}^{2}-\lambda_{p}{∥{\tilde{x}}_{p}\left(k\right)∥}^{2}.$$

Then, the equilibrium of the complete ESynPID topology is locally asymptotically stable. Therefore,

(A29) $$\lim_{k\to \infty }e_{j}\left(k\right)=0,\;j\in \left\{1,2,3\right\},$$

and, in particular,

(A30) $$\lim_{k\to \infty }\left(y_{j}\left(k\right)-r_{j}\left(k\right)\right)=0,\;j\in \left\{1,2,3\right\}.$$

Moreover, the inverter state also converges in the nominal case:

(A31) $$\lim_{k\to \infty }\xi_{r}\left(k\right)=0,$$

with respect to the equilibrium defined by the desired target values and the steady-state feedback perfusion.

Hence, the architecture shown in Figure 1, composed of the PID Inversor, Error Generator, controller, Open Loop Modulator, control-to-range module, Phase Switching mechanism, pump emulation block, S/H stages, and Virtual Patient, is locally asymptotically stable in the nominal case and practically stable in the presence of bounded disturbances and implementation constraints.
